# Visual Analysis of Transcriptome Data in the Context of Anatomical Structures and Biological Networks

**DOI:** 10.3389/fpls.2012.00252

**Published:** 2012-11-15

**Authors:** Astrid Junker, Hendrik Rohn, Falk Schreiber

**Affiliations:** ^1^Leibniz Institute of Plant Genetics and Crop Plant Research GaterslebenGatersleben, Germany; ^2^Institute of Computer Science, Martin Luther University Halle-WittenbergHalle, Germany; ^3^Clayton School of Information Technology, Monash UniversityClayton, VIC, Australia

**Keywords:** omics data visualization, expression atlas, data integration, color-coding, biological network, systems biology graphical notation, visual analytics

## Abstract

The complexity and temporal as well as spatial resolution of transcriptome datasets is constantly increasing due to extensive technological developments. Here we present methods for advanced visualization and intuitive exploration of transcriptomics data as necessary prerequisites in order to facilitate the gain of biological knowledge. Color-coding of structural images based on the expression level enables a fast visual data analysis in the background of the examined biological system. The network-based exploration of these visualizations allows for comparative analysis of genes with specific transcript patterns and supports the extraction of functional relationships even from large datasets. In order to illustrate the presented methods, the tool HIVE was applied for visualization and exploration of database-retrieved expression data for master regulators of *Arabidopsis thaliana* flower and seed development in the context of corresponding tissue-specific regulatory networks.

## Introduction

The development of complex multicellular plant structures and the coordination of plant growth in response to external and internal stimuli are the result of elaborate gene activity patterns in space and time. Transcriptomics, as the large- or genome-scale profiling of all transcripts in a biological sample (representing a cell, tissue, or organ) is established as standard technique in functional genomics and molecular biology with a wide spectrum of corresponding analytical methods. Basically three different approaches are discriminated: (1) hybridization-based methods (microarrays, tiling arrays), (2) PCR-based methods (quantitative real-time PCR), and (3) sequencing-based methods (RNA Sequencing). Bioanalytical advances and the accompanying decrease of costs for high-throughput technologies led to the generation of comprehensive transcriptomics datasets spanning multiple developmental stages, as well as different tissues and organs. Furthermore, sampling techniques such as laser microdissection (Nelson et al., 2006; Day, 2010) allow the precise isolation of very small amounts of tissue thereby substantially increasing the resolution of transcriptomics analyses from the level of small tissues (Cai and Lashbrook, 2008; Brooks et al., 2009; Matas et al., 2011; Endo et al., 2012) to the level of single cells (Nelson et al., 2008; Schmidt et al., 2011; Thiel et al., 2012; Yang et al., 2012). As a result of increasing complexity and resolution transcriptomics datasets are able to cover the whole development of plant organs in its multitude of spatial and temporal dimensions such as the transcriptomic landscape of *Arabidopsis* seed development (Le et al., 2010) and the atlas of *Arabidopsis* root gene expression (Birnbaum et al., 2003).

Due to publishers requests an increasing number of transcriptome datasets is incorporated into public transcriptomics data repositories which have varying complexity with regard to covered species and provide different sets of functionalities for data analysis and visualization. These resources represent an important complementary pool of information supporting researchers in comparative data interpretation and gain of knowledge in the context of the “global” expertise. Analyzing the wealth of transcriptomics datasets (either from own experiments or database-retrieved datasets or in combination), the challenge is to identify sets of genes of interest following a particular pattern of expression. Besides corresponding statistical tests and clustering procedures, appropriate data visualization techniques play a pivotal role in these analyses with the requirement of presenting complex and multi-dimensional datasets in a clear and cohesive manner. Numerous tools have been developed for network in- or dependent visualization of expression profile datasets (Suderman and Hallett, 2007; Gehlenborg et al., 2010). The most common way of network-independent visualizing expression profiles are heatmaps as a color-coding-based representation of expression values in a matrix-like format spanning genes and conditions. In order to complement these visualization-based approaches, the integration of data into biological networks providing contextual process information enhances the detection of functional relationships (e.g., the identification of functional modules by integrating transcriptomics datasets into gene-regulatory networks). Network-dependent visualization are generated by adjusting node attributes (color, shape) according to the expression value (such as in, e.g., Cytoscape, Shannon et al., 2003 or Ondex, Kohler et al., 2006) or by visualizing more complex charts, such as bar or line charts inside the network nodes as supported by VANTED (Junker et al., 2006) or PathVisio (Van Iersel et al., 2008). Spatial information is neglected so far and the integration of transcriptomics data into 2D images is not supported by the available computational tools.

Here we propose the visualization and exploration of multi-dimensional transcriptomics datasets in the context of the underlying anatomical structures, which in fact results in complex spatial “diagrams” represented on image data describing the examined biological system. This method combines structural information, and quantitative gene expression data in their spatio-temporal context thereby providing an intuitive and compact visualization for enhanced visual analysis of large-scale expression analyses. The tool HIVE (Rohn et al., 2011) enables biologists to integrate 2D images, expression data, and biological networks, with the underlying VANTED framework (Junker et al., 2006) offering further basic network editing and data visualization functionalities. The proposed method is transferable to all -omics domains, thus can be applied for visualization of e.g., metabolomics data in the context of anatomical structures and metabolic networks. In the following we illustrate the technical background as well as the visualization and exploration aspects of the proposed methodology. We demonstrate its applicability by visualizing database-retrieved expression values of transcription factor genes implicated with *Arabidopsis thaliana* flower and seed development in the context of the corresponding anatomical structures and by integration of these images into organ-specific gene-regulatory networks. Finally, we discuss the advantages of the methodology and further applications.

## Methods

**Figure 1:**
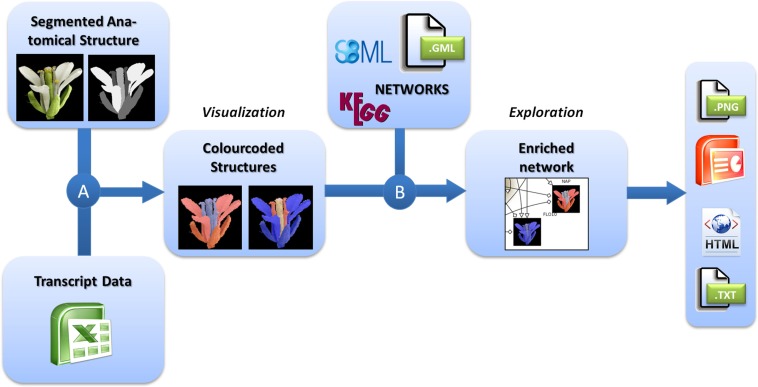
**Workflow for the visualization of transcriptomics data on images in the context of biological networks**. **(A)** Transcriptomics and 2D images of anatomical structures are integrated by color-coding of image segments according to the respective transcript data. **(B)** These color-coded images are integrated into nodes of biological networks. Such integrated views can be explored interactively and exported in various formats for individual purposes.

For the integrated visualization and exploration of transcriptome data in the context of anatomical structures and biological networks, the user has to follow a two-step procedure comprising two consecutive integration steps (Figure 1). In the first step, expression values (e.g., measured for different tissues of an organ) are assigned to image segments corresponding to the biological structures they represent. Image segments are color-coded on the basis of different expression values. In a second step, color-coded images representing the expression profiles of different genes are integrated into the corresponding nodes of a biological network. Two examples using this workflow are described in the Application section and a step-by-step protocol for using the HIVE tool is given in File S1 in Supplementary Material.

### Integration of transcriptomics data and 2D images

For the visualization of transciptomics data in the context of anatomical structures (Figure 1A), the user first needs to import the transcriptome dataset into HIVE as well as the segmented 2D image representing biological structures that have been the basis for transcriptome analysis.

HIVE offers a template-based import for numerical data where normalized expression values are uploaded together with experiment meta-information such as project name, investigated subjects, and conditions as well as spatial identifiers which link numerical values with the different image segments. Any kind of transcriptome dataset can be transferred into the HIVE template and used for integration into images, regardless of data source or level of complexity/resolution regarding multiple considered conditions, organs, or timepoints. A list of publically accessible plant transcriptome data resources is given in Table 1.

**Table 1:** **Plant transcriptome databases**.

Database	Plant species	Link	Reference
**CROSS-SPECIES RESOURCES**			
Array express	∼	http://www.ebi.ac.uk/arrayexpress/	Brazma et al. (2003)
Co-expressed biological processes (COP)	*Arabidopsis thaliana*, *Populus trichocarpa*, *Oryza sativa*, *Hordeum vulgare*, *Zea mays*, *Glycine max*, *Triticum aestivum*, *Vitis vinifera*	http://webs2.kazusa.or.jp/kagiana/cop/	Ogata et al. (2009)
Gene expression omnibus (GEO)	∼	http://www.ncbi.nlm.nih.gov/geo/	Edgar et al. (2002)
Genevestigator	*Arabidopsis thaliana*, *Hordeum vulgare*, *Oryza sativa*, *Triticum aestivum*, *Nicotiana tabacum*, *Physcomitrella patens*, *Solanum lycopersicum*, *Zea mays*	https://www.genevestigator.com/gv/	Zimmermann et al. (2004)
NASCArrays	∼	http://affymetrix.arabidopsis.info	Craigon et al. (2004)
Stanford microarray database (SMD)	∼	http://smd.stanford.edu/	Sherlock et al. (2001)
The botany array resource (eFP browser)	*Arabidopsis thaliana*, *Populus trichocarpa*, *Medicago truncatula*, *Oryza sativa*, *Hordeum vulgare*, *Zea mays*, *Glycine max*, *Solanum tuberosum*	http://bar.utoronto.ca/welcome.htm	Winter et al. (2007)
**SPECIES-SPECIFIC RESOURCES**			
*Arabidopsis* transcriptome genomic express database	*Arabidopsis thaliana*	http://signal.salk.edu/cgi-bin/atta	Yamada et al. (2003)
CSB.DB	*Arabidopsis thaliana*	http://csbdb.mpimp-golm.mpg.de/	Steinhauser et al. (2004)
Maize C3/C4 transcriptomic database	*Zea mays*	http://c3c4.tc.cornell.edu/search.aspx	–
*Medicago truncatula* gene expression atlas	*Medicago truncatula*	http://mtgea.noble.org/v2/	Benedito et al. (2008)
PopGenIE	*Populus trichocarpa*	http://www.popgenie.org/	Sjodin et al. (2009)
RiceGE	*Oryza sativa* (indica/japonica)	http://signal.salk.edu/cgi-bin/RiceiGE; http://signal.salk.edu/cgi-bin/RiceGE	Li et al. (2006), Liu et al. (2007)
RNA-seq atlas of *Glycine* max	*Glycine max*	http://soybase.org/soyseq/	Severin et al. (2010)
Tomato expression database	*Solanum lycopersicum*	http://ted.bti.cornell.edu/	Fei et al. (2006)
Transcriptome atlas of *Glycine* max	*Glycine max*	http://digbio.missouri.edu/soybean_atlas/	Libault et al. (2010)

For integration of transcript data and 2D image, a segmentation step needs to be performed in order to assign image pixels to all measured tissues, conditions, or developmental stages. Different segments of the image may represent different tissues of an organ but also different developmental stages or even similar anatomical structures for the display of varying experimental conditions or a set of different plant lines. This is achieved by concatenating the images to one montage image of, e.g., different stages or conditions and repeating the process of segmentation for all segments of different stages. During segmentation every image region of interest (e. g., a certain tissue or cell type) is filled by a unique color which is representative for this part of the anatomical structure. The resulting image (called “labelfield” image) has to be saved as a second image accompanying the source image. For segmentation the user is referred to simple paint tools such as Microsoft Paint, more advanced graphics tools such as GIMP^1^ and Adobe Photoshop^2^ and specialized segmentation software such as ImageJ (Abramoff et al., 2004) and Amira (Stalling et al., 2005; for a review about biological image data and segmentation see (Walter et al., 2010). During integration, HIVE creates an image for each gene of the dataset by setting all pixels of a segment (representing a certain tissue, stage, or experimental condition) according to the respective expression value. A global color-map assigns the transcript value measured in the anatomical structures at a specific stage to red (high expression) or blue (low expression) dye.

The resulting color-coded images represent the expression profiles of genes in a biological intuitive way and, in contrast to looking at tables or heatmaps, facilitate substantially the biological understanding and comparative analysis. Even for a large number of genes of interest, this visualization enables the fast detection of interesting expression patterns at a glance and in a high-throughput manner. Visual screening enables to detect even detailed facts from an overview perspective. In order to reduce the complexity of a dataset, HIVE offers the possibility to perform condition-dependent expression analyses by switching between images for different conditions or by visualizing relative expression values calculated as the difference between different conditions. Additional manual rearrangement of images with similar patterns represents an intuitive way of visual clustering thereby taking into account biological prior knowledge. Finally, static and comprehensive visualizations of a rather complex system can be easily published in the web, publications, or used in talks in order to communicate gained knowledge. HIVE supports the export of such visualizations as high-resolution raster images such as JPG and PNG, as well as vector graphics such as SVG, PDF, and PPT. Furthermore, interactive visualizations can be automatically generated from the active set of open networks and posted as a website containing a gallery of images (Junker et al., 2012). The network nodes of the images may contain hyperlinks to other networks as well as to external websites such as web-based database entries and thereby enable the interactive browsing of achieved results in the context of linked resources.

### Integration of color-coded images and biological networks

Multi-domain -omics data builds the basis for the generation of biological networks (Moreno-Risueno et al., 2010) with the network type (signaling, metabolic, gene-regulatory, protein–protein-interaction) depending on afore measured type of data. As the basis for various modeling approaches and as a framework for visualization of -omics data biological networks are well established as systems biology resource. For plants, a huge number of computationally derived and manually curated networks of different types exists (Table 2). HIVE supports the exchange of networks using standard file formats such as GML, SBML, and BioPAX (Table 3). As part of the VANTED framework, HIVE is connected to functionalities of the SBGN-ED tool (Czauderna et al., 2010) and therefore provides support for the Systems Biology Graphical Notation (SBGN, Le Novere et al., 2009). Similar to wiring diagrams in engineering, SBGN standardizes the representation of biological networks using a well-defined semantics and small set of easily recognizable glyphs. This unambiguous way of network visualization allows for an efficient transfer of knowledge among the biologist community and facilitated access to the growing number of SBGN-supporting web resources.

**Table 2:** **Web resources for plant networks**.

Network	Species	Link	Standards	Reference
**GENE-REGULATORY NETWORKS**				
AGRIS	*Arabidopsis thaliana*	http://arabidopsis.med.ohio-state.edu/REIN/	CSV*	Yilmaz et al. (2011)
Regulog	*Arabidopsis thaliana*	http://interolog.gersteinlab.org	CSV*	Yu et al. (2004)
RIMAS	*Arabidopsis thaliana*	http://rimas@ipk-gatersleben.de	SBGN GML	Junker et al. (2010)
**METABOLIC NETWORKS**				
KEGG	*Arabidopsis lyrata*, *Arabidopsis thaliana*, *Oryza sativa*, *Populus trichocarpa*, *Ricinus communis*, *Sorghum bicolor*, *Vitis vinifera*, *Zea mays*	http://www.genome.jp/kegg/pathway.html	KGML	Ogata et al. (1999), Kanehisa et al. (2010), Kanehisa et al. (2012)
MetaCrop	*Arabidopsis thaliana*, *Beta vulgaris*, *Brassica napus*, *Hordeum vulgare*, *Medicago truncatula*, *Oryza sativa*, *Solanum tuberosum*, *Triticum aestivum*, *Zea mays*	http://metacrop.ipk-gatersleben.de/	SBML SBGN GML	Schreiber et al. (2012)
PANTHER	*Arabidopsis thaliana*, *Oryza sativa Japonica*	http://www.pantherdb.org/pathway/	SBGN BioPAX	Mi et al. (2010)
PlantCyc	*Arabidopsis thaliana*, *Brachypodium distachyon*, *Oryza sativa*, *Populus trichocarpa*, *Sorghum bicolor*, *Zea mays*	http://plantcyc.org/	BioPAX	Caspi et al. (2006)
Reactome	*Arabidopsis thaliana*, *Brachypodium distachyon*, *Glycine max*, *Medicago trunculata*, *Nicotiana tabacum*, *Oryza sativa*, *Populus trichocarpa*, *Sorghum bicolor*, *Vitis vinifera*, *Zea mays*	http://www.reactome.org/	SBGN SBML BioPAX	Croft et al. (2011)
Wiki pathways	*Arabidopsis thaliana*, *Oryza sativa*, *Zea mays*	http://www.wikipathways.org	BioPAX	Kelder et al. (2012)
**CO-EXPRESSION NETWORK**				
AraNet	*Arabidopsis thaliana*, *Hordeum vulgare*, *Oryza sative*, *Populus trichocarpa*, *Triticum aestivum*, *Medicago truncatula*, *Glycine max*	http://aranet.mpimp-golm.mpg.de/aranet/	CSV*	Mutwil et al. (2010, 2011)
AGCN	*Arabidopsis thaliana*		SIF	Mao et al. (2009)
atGGN	*Arabidopsis thaliana*	http://bioinformatics.cau.edu.cn/atGGN/	WWW	Ma et al. (2007)
@CoEX	*Arabidopsis thaliana*	http://ibis.tau.ac.il/AthMod/	WWW	Atias et al. (2009)
ATTED-II	*Arabidopsis thaliana*, *Oryza sativa*	http://atted.jp	CSV*	Obayashi et al. (2009), Obayashi et al. (2011)
Gene co-expression network browser	Maize, rice	http://www.clemson.edu/genenetwork/network.php	WWW	Ficklin and Feltus (2011)
Co-expressed gene network in barley	*Hordeum vulgare*	http://coexpression.psc.riken.jp/barley/index.pl	CSV*	Mochida et al. (2011)
**PROTEIN–PROTEIN-INTERACTION NETWORK**				
AtPIN	*Arabidopsis thaliana*	http://bioinfo.esalq.usp.br/atpin	SIF	Brandao et al. (2009)
Biogrid	*Arabidopsis thaliana*, *A. lyrata*, *Brachypodium*, *Glycine max*, *Oryza sativa*, *Populus trichocarpa*, *Sorghum bicolor*, *Zea mays*	http://thebiogrid.org/	CSV*	Stark et al. (2006)
IntAct	*Arabidopsis thaliana*, *Oryza sativa*	http://www.ebi.ac.uk/intact/	CSV*	Kerrien et al. (2012)
Interolog	*Arabidopsis thaliana*	http://interolog.gersteinlab.org/	CSV*	Yu et al. (2004)
iRefIndex	*Arabidopsis thaliana*, *Arachis hypogaea*, *Artocarpus heterophyllus*, *Artocarpus integer*, *Canavalia ensiformis*, *Glycine max*, *Hordeum vulgare*, *Nicotiana tabacum*, *Oryza sativa*, *Oryza sativa Japonica*, *Petunia x hybrid*, *Pisum sativum*, *Solanum lycopersicum*, *Spinacia oleracea, Triticum aestivum*, *Zea mays*	http://irefindex.uio.no/wiki/iRefIndex	CSV*	Razick et al. (2008)
MiMI	*Arabidopsis thaliana*, *Oryza sativa* Japonica	http://mimi.ncibi.org/MimiWeb/main-page.jsp	CSV*	Jayapandian et al. (2007)
PAIR	*Arabidopsis thaliana*	http://www.cls.zju.edu.cn/pair/	CSV*	–
Pathway commons	*Arabidopsis thaliana*	http://www.pathwaycommons.org/pc/home.do	BioPAX SIF	Cerami et al. (2011)
String	*Arabidopsis lyrata*, *A. thaliana*, *Brachypodium distachyon*, *Oryza sativa Indica* Group, *O. sativa Japonica* Group, *Populus trichocarpa*, *Sorghum bicolor*, *Vitis vinifera*	http://string-db.org	WWW	Szklarczyk et al. (2011)
**SIGNAL TRANSDUCTION PATHWAYS**				
PANTHER	*Arabidopsis thaliana*, *Oryza sativa Japonica*	http://www.pantherdb.org/pathway/	SBGN BioPAX	Mi et al. (2010)
Pathway commons	*Arabidopsis thaliana*	http://www.pathwaycommons.org/pc/home.do	BioPAX SIF	Cerami et al. (2011)
Wiki pathways	*Arabidopsis thaliana*, *Oryza sativa*, *Zea mays*	http://www.wikipathways.org	BioPAX	Kelder et al. (2012)

*Requires preprocessing in order to get a network for upload in HIVE, WWW indicates webresources without the possibility for download of network files.

**Table 3:** **Common network file formats (supported by HIVE)**.

	Description	Reference/Link
BioPAX (Biological pathway exchange)	Standardized exchange format of various network editing tools, which supports all graph attributes, such as topology, layout, visual properties, links, and also biological properties (e.g., roles, functions); hard to edit manually	Demir et al. (2010)
CSV* (Comma separated values)	Text-based exchange format of some network editing tools, which supports basic network topology without any layout information; easy to edit manually with text editors or MS Excel	–
GML (Graph modeling language)	Standardized text-based exchange format of various network editing tools, which supports all graph attributes, such as topology, layout, visual properties, links, and experiment data; hard to edit manually	http://www.fim.uni-passau.de/fileadmin/files/lehrstuhl/brandenburg/projekte/gml/gml-technical-report.pdf
SBGN (SBGN markup language, SBGN-ML)	Standardized XML-based format for the exchange of SBGN maps, which so far supports only the exchange of basic network topology without any layout information; hard to edit manually	Van Iersel et al. (2012); http://libsbgn.sourceforge.net
SIF (Simple interaction file)	Text-based exchange format of various network editing tools, which is similar to CSV and supports basic network topology without any layout information; easy to edit manually with text editors or MS Excel	Shannon et al. (2003)
SBML (Systems biology markup language)	Standardized XML-based exchange format of various tools for representing biochemical models, which supports biological properties (e.g., roles, functions) and basic network topology without any layout information; hard to edit manually	Hucka et al. (2003)

The integration of color-coded anatomical structures and networks in HIVE relies on data mapping depending on equal gene identifiers in the network and the transcriptome dataset, the latter of which have been assigned to the color-coded images in the first integration step. As a result the color-coded images are visualized inside the corresponding network nodes (Figure 1B). In addition to data integration, HIVE and the underlying VANTED framework support the exploration of such complex visualizations combining numerical data, images, and biological networks by providing various functionalities for zooming, panning, and advanced exploration techniques such as collapsing sub-graphs. In addition to the static visualization of color-coded images inside network nodes, interactive exploration enables the user to investigate color-coded images in the side-panel when hovering over or selecting the respective network nodes whereas the selection of multiple nodes results in a stacked visualization of respective color-coded images in the side-panel. This interaction technique, called brushing (Martin and Ward, 1995) allows for enlarging and highlighting the expression profiles of selected genes and is applied for stepwise exploration of comprehensive datasets.

## Applications

### Usecase 1: Visual analysis of floral homeotic gene expression patterns in the context of a gene-regulatory network

**Figure 2:**
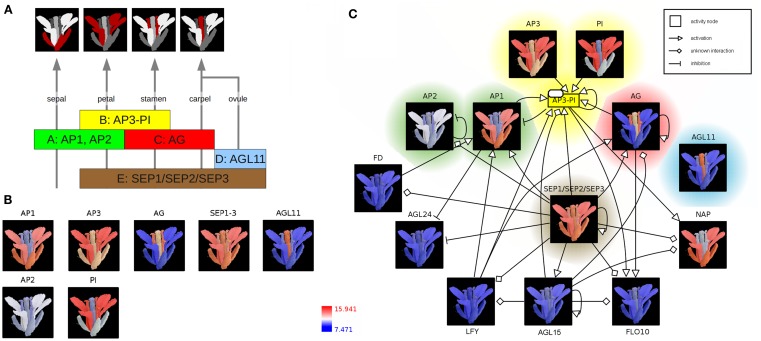
**The ABC(DE)-model of *Arabidopsis thaliana* floral organ specification**. **(A)** Determination of floral organ identity depends on the combinatorial expression of floral homeotic genes from different classes. **(B)** Color-coding of an *A. thaliana* flower image based on expression values (red: high expression; blue: low expression) **(C)** Integration of color-coded images, representing floral homeotic gene expression patterns, into the context of a regulatory network. The network is represented using the Activity Flow (AF) language of SBGN.

According to the well-known ABC(DE)-model floral organ specification is dependent on the combinatorial expression of different members of the five classes of floral homeotic genes (Figure 2A; Coen and Meyerowitz, 1991; Weigel and Meyerowitz, 1994; Theissen and Saedler, 2001). Genetic studies of corresponding mutants (especially in *A. thaliana*) identified the class A genes *APETALA1* (*AP1*) and *APETALA2* (*AP2*) in determining sepal and petal identity, the latter in combination with class B genes *APETALA3* (*AP3*) and *PISTILLATA* (*PI*) and a member of the class E genes *SEPALLATA* (*SEP1–3*). Co-expressed with the C-class gene *AGAMOUS* (*AG*), these B and E class genes specify stamen identity whereas the expression of *AG* and *SEP* leads to the development of the carpel. Ovule identity is determined by the expression of a *SEP* gene together with the class D gene *AGAMOUS-LIKE11* (*AGL11*). Finally, homeotic proteins interact to form different heterotetrameric complexes, known as the floral quartets (Theissen and Saedler, 2001).

In the following, expression profiles of *Arabidopsis* homeotic genes are visualized as colored image segments of the *Arabidopsis* flower in the context of a gene-regulatory network. Expression values for floral homeotic genes were downloaded from the Genevestigator database (Zimmermann et al., 2004; Schmid et al., 2005) and parsed into the HIVE Excel template file. Furthermore, an electron microscopy image of a mature *A. thaliana* flower was manually segmented discriminating the four main floral whorls sepal, petal, carpel (including ovules), and stamen and imported into the tool (File S2 in Supplementary Material). In the first integration step, expression values are mapped to the image segments which are then color-coded depending on the level of expression (Figures 1A,B). The resulting colored images provide an intuitive visualization of the investigated gene expression profiles in the context of the biological system “flower” and clearly demonstrate the role of the distinct homeotic genes in floral organ determination. Most expression profiles show the expected, stereotyped patterns (Figure 2B). The visualized expression dataset does only consider the mature flower stage (21d of *Arabidopsis* development, Schmid et al., 2005) which might be the reason for deviating expression patterns due to changing expression levels of homeotic genes at different stages of flower development. *AP1* (class A) is expressed in sepals and petals whereas *AP2* (class A) is known to be expressed at the earlier stages of flower development (Wollmann et al., 2010) and therefore missing in mature sepals. *AP3* as well as *PI* (class B) show the expected high expression levels in stamen and petals, but also lower expression in other organs. *AG* (gene class C) is solely expressed in stamen and carpel. *AGL11* does not show any regulatory interactions with the other homeotic genes and is depicted here as D class representative the expression of which can be found exclusively in the ovula (carpel). *SEP1*, *2*, and *3* (class E) are functionally redundant genes (Pelaz et al., 2000), which are active in all flower organs and have a large regulatory influence on the development of the floral context, except sepals (Flanagan and Ma, 1994).

In order to perform the second integration step (Figure 1B), regulatory interactions of *AP1* and *AP3* were retrieved from AGRIS (Yilmaz et al., 2011), imported into HIVE, manually merged and translated into valid SBGN Activity Flow style (Huaiyu et al., 2009; File S2 in Supplementary Material). Subsequently, color-coded images were mapped to the correspondent network nodes thereby setting spatial expression profiles into the context of homeotic gene-regulatory interactions (Figure 2C, File S2 in Supplementary Material). Nodes are highlighted according to the colors of the respective gene class in Figures 2A,B. The *SEP1*, *2*, *3* genes (class E) seem to be a central hub in the homeotic gene-regulatory network underpinning their global function for the floral context. Their unspecific expression in all four whorls is important in positive feedback loops securing the expression of whorl-specific homeotic genes at later stages of flower development (Liu and Mara, 2010).

The network additionally comprises transcription factors (TF) which act as regulators of homeotic gene expression (Figure 2C) such as LEAFY (LFY) acting as key player in the switch from vegetative to reproductive development (Wagner et al., 1999). The activation of *AP1* by LFY takes place during floral initiation. As regulatory interactions in the network (Figure 2C) are shown independent of any developmental timepoint, the expression profiles allow for drawing conclusions about stage- or tissue-specific regulatory interactions. In accordance with this, the missing co-expression of *LFY* and *AP1* in the mature flower (Figure 2C, LFY not expressed, blue color code) indicates that the corresponding interaction takes place at another stage of flower development.

### Usecase 2: Visual analysis of *Arabidopsis* seed expression profiles with developmental and spatial resolution

**Figure 3:**
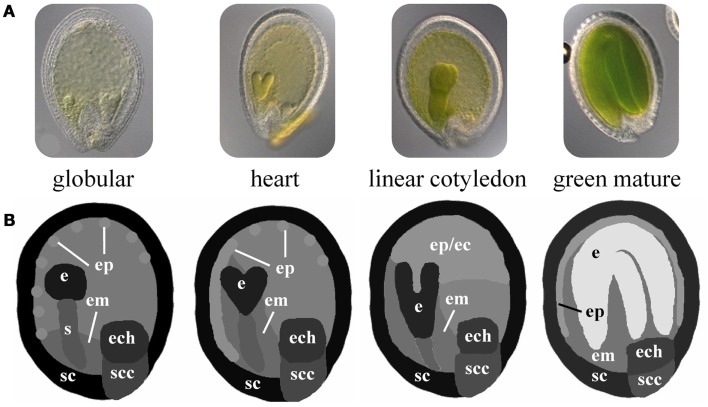
**Schematic 2D images of different stages of *Arabidopsis* seed development**. **(A)** Microscopic images of *Arabidopsis* seeds at the globular, heart, linear cotyledon, and green mature stage. **(B)** Corresponding not-to-scale schematic representations of the four seed stages which have been used for integration of transcriptome data. (e, embryo; s, suspensor; em, endosperm micropylar; ep, endosperm peripheral; ec, endosperm cellularized; ech, endosperm chalazal; sc, seed coat; scc, seed coat chalazal.)

*Arabidopsis* seed development is a well studied developmental process comprising morphogenetic processes during the early stages (embryo morphogenesis) and physiological processes such as storage compound accumulation and acquisition of desiccation tolerance during seed maturation preparing the seed to survive unfavorable conditions and to nourish the growing embryo after germination. Independent of level and specificity, the expression of genes during seed development implies their functional relevance in any of the underlying morphogenetic and physiological processes. Especially for genes with regulatory functions, which need to have a large combinatorial interaction potential in order to assure proper development, it is important to identify co-expressed genes and connected functional modules in all spatial and temporal dimensions. Le and co-workers (Le et al., 2010) performed a global (genome-scale) transcriptome analysis of *Arabidopsis* seed development with high spatial and developmental resolution considering five developmental stages (preglobular, globular, heart, linear cotyledon, and green mature stage) with each seven seed tissues (embryo, suspensor, micropylar/chalazal/peripheral endosperm, seed coat, and chalazal seed coat). A part of this dataset will be used in the following in order to illustrate the presented method. Using the Excel template, expression values for 400 regulatory genes (e.g., transcription factors, TF) have been imported into HIVE (File S2 in Supplementary Material) and used for integration into the respective seed 2D images (Figure 1A). These have been adapted from light microscopical images of staged *Arabidopsis* seeds. The proportions of the segmented seed tissues are not-to-scale in order to facilitate the visual analysis of tissues with size differences (such as globular stage embryo in comparison to the mature endosperm, Figure 3). The use of schematic representations of the anatomical structures enables to adjust tissue proportions for enhanced visual analysis without losing the biological context information. Images for all four seed stages were combined into one montage image and imported into HIVE (File S3 in Supplementary Material).

**Figure 4:**
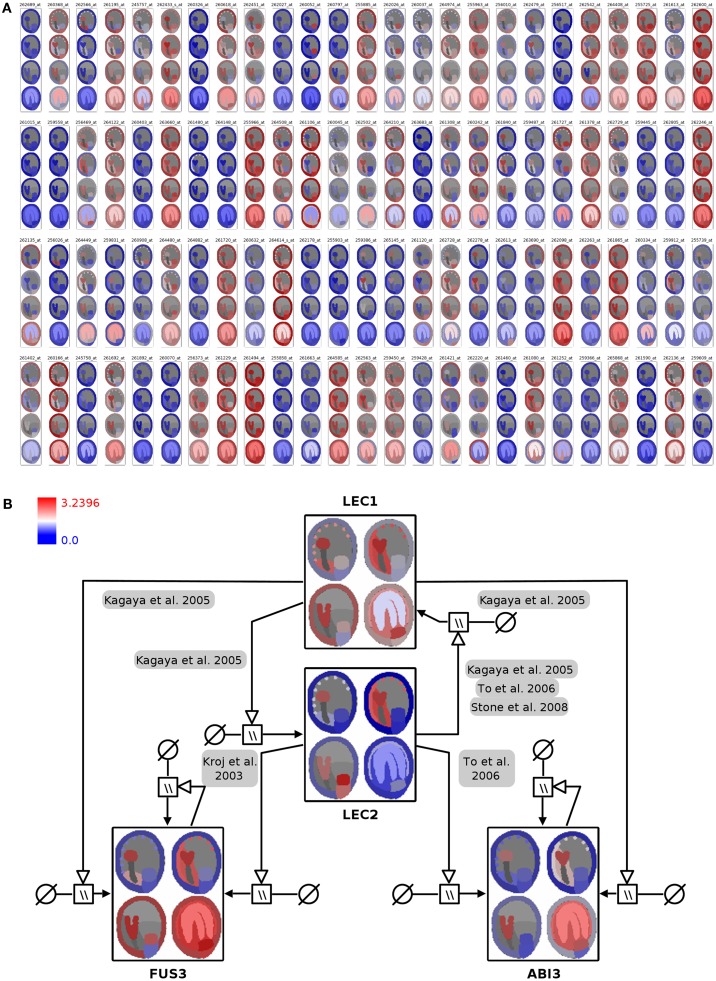
**Visualization of seed regulator expression profiles with spatio-temporal resolution in the context of the seed anatomical structure and gene-regulatory network**. **(A)** Seed expression profiles of 100 *Arabidopsis* genes with regulatory functions, using 2D seed images for display of the corresponding spatio-temporal resolution (red: high expression; blue: low expression). **(B)** Integration of color-coded seed images into the LEC1/AFLB3 regulatory network. LEC1 seems to function upstream of LEC2, FUS3, and ABI3 (Meinke et al., 1994; Kagaya et al., 2005; To et al., 2006; Stone et al., 2008) whereas LEC2 in turn controls FUS3 and ABI3 (Kroj et al., 2003; To et al., 2006). During linear cotyledon and green mature stages expression of *LEC1* and *LEC2* ceases and expression levels of *FUS3* and *ABI3* stay constant due to autoregulatory loops (Kroj et al., 2003; To et al., 2006). For detailed explanations about the used SBGN Process Descriptions glyphs the reader is referred to (Junker et al., 2010). Please note that the arrangement of the four seed images was adapted from a vertical row in **(A)** to a 2 × 2 matrix arrangement in **(B)** for layout purposes.

In the first integration step the expression profiles of 400 genes were mapped onto the image representing four stages of seed development in tissue resolution (Figure 4A, full resolution image in File S3 in Supplementary Material). On the basis of this visualization, visual clustering, and screening enable the fast exploration and interpretation of the dataset and allow for selection of genes with specific expression patterns (e.g., stage- or tissue-specific expression).

In *Arabidopsis* four genetic loci, *LEAFY COTYLEDON1* (*LEC1)*, *LEAFY COTYLEDON2* (*LEC2)*, *FUSCA3* (*FUS3)*, and *ABSCISIC ACID INSENSITIVE3 (ABI3)* are considered to regulate main processes of embryogenesis and seed maturation (for review see Santos-Mendoza et al., 2008). The four TF constitute a network of distinct but partially redundant pathways which have been analyzed by the genetic, molecular, and phenotypic characterization of single and multiple mutants (To et al., 2006). The corresponding manually curated gene-regulatory network in the SBGN Process Description language (Moodie et al., 2011) was derived from the RIMAS web portal (Junker et al., 2010). The expression profiles of the four TF were integrated as color-coded seed images into the respective network nodes (Figure 4B; File S3 in Supplementary Material). This integrated graph allows for a fast comparative visual analysis of the expression patterns of TF in the context of a regulatory cascade with LEC1 being hierarchical superior in comparison to the remaining three factors. *LEC1* and *LEC2* expression is mainly occurring during the early seed stages. Similar expression patterns of *ABI3* and *FUS3*, with respect to expression in the embryo and the micropylar endosperm, indicate that regulatory interactions between these factors take place during early stages. The expression of *ABI3* and *FUS3* in the later stages of seed development is secured by positive autoregulatory loops.

## Discussion

The present approach for integrative visualization of multimodal abstract data provides interactive, visual representations of data to amplify cognition and follows typical recommendations regarding the design of *Information Visualization* (InfoVis) applications (Card et al., 1999; Carr, 1999; Ward et al., 2010). It provides compact graphical representations of large datasets, which enable the scientist to discover interesting patterns or functional relationships.

The integration of three types of biological data (expression values, 2D images, and networks) extends former integration approaches by taking into account spatial information. The integration of transcriptomic datasets and images (2D structures) has been largely neglected so far, although an image is the best way of transferring knowledge as “it can do for the mind what automobiles can do for the feet” (Card et al., 1999). We use images as a medium for communicating relevant information which might otherwise be concealed in the complexity of transcriptome datasets. This approach facilitates the interpretation of complex datasets since it relieves the biologist of associating the initial biological structure with otherwise abstract data points in all possible dimensions. To our knowledge, HIVE is the first tool supporting the visualization of spatio-temporally resolved expression datasets by color-coding of images representing the examined anatomical structure in a customized and semi-automatic way.

The only similar approach is offered by the eFP web resource visualizing transcriptome data by color-coding of electronic pictographs (Winter et al., 2007). The eFP browser has been adapted to a series of plant species and developmental stages each represented by the respective set of pictographs with down to cellular resolution for certain organs such as roots. In addition it covers transcriptome analyses derived from stress experiments and a series of different plant treatments. As an online resource it is very comprehensive, easy to handle, and provides a large set of visualizations which are very useful for comparative analysis with own data. Although facilitating browsing expression profiles of the gene(s) of interest, it does not provide the possibility to visualize own (user-derived) data which would require adapting the visualization to individual purposes such as individual transcriptome datasets, individual experimental setups (conditions) or the resolution of the examined biological system and the corresponding image segments. Compared to the eFP browser, the HIVE tool with the presented functionalities offers full customization with regard to the analyzed biological system (any species), the resolution of the measurement data in space and time (any conditions, time, or developmental series at organ-, tissue-, or cellular-level as represented by the image), the type of numerical data (metabolite measurements, transcript profiles, proteome data) and multiple visualization options. HIVE additionally allows for the integration of color-coded images into networks, a feature which is not supported by the eFP browser.

The first step of the HIVE integration workflow (Figure 1A) has a few hands-on requirements such as manual image segmentation before import into the tool and the assignment of segments to the corresponding tissues/cell types during integration. The resulting images (Figures 2B and 4A) provide a cohesive and unprecedented overview of large-scale transcriptomics datasets which is fully customizable to any level of spatio-temporal resolution and enables fast visual analysis. In terms of scalability, currently it is possible to visualize and explore several hundred nodes, depending on the image size. For high numbers of genes, it might be necessary to reduce the dataset by isolating and examining context-specific parts of the dataset, which can be realized using different HIVE functionalities for condition-dependent analyses or by manually rearranging or deleting genes in order to extract relevant knowledge. This could be further enhanced through the extension of HIVE by automated image-based clustering. Furthermore, images with integrated expression data could serve as the basis for the generation of co-expression networks with edges of varying thickness representing the level of co-expression of two genes.

The second integration step (Figure 1B) performs an automatic mapping of the color-coded images to respective nodes of a custom network with HIVE supporting various standard network files. Although both use cases in the present manuscript integrate expression data into gene-regulatory networks, there are no combinatorial limitations regarding network types. Visual analysis of the corresponding integrated graphs facilitates the extraction of knowledge by for example: (a) visually identifying functional relationships which are specifically occurring in certain tissues or cell types (as represented by image segments), (b) visually identifying heterochronic effects in gene expression developmental/time series in the context of functional relationships, or (c) simplifying the comparative visual analysis of expression profiles of connected genes such as transcriptional co-regulators or regulators and target genes. Furthermore, the integration of transcriptome data into networks provides a temporal or developmental context for the functional relationships. The integration of more complex datasets (comprising several stages or different lines such as wild type-mutant comparisons) enables the user to specify stage-specific regulatory networks or even to derive new regulatory interactions which then have to be verified using wet lab methods.

In general the proposed method is applicable to the visualization of any kind of numerical data (proteomics, metabolomics data) independent of the applied experimental methods or the examined biological system (model species). It only requires an image or image montage representing all dimensions (conditions, genotypes, developmental stages) of the dataset, which should not be problematic with respect to the current advances in microscopical techniques or by using schematic representations of the underlying anatomical structures.

## Conclusion and Outlook

In summary, the presented approach provides a way for cohesive visualization of complex biological datasets in combination with complementary biological information (networks, images). The method does not have any restrictions with respect to the examined biological system, data source, or experimental methods used for data acquisition. It provides the possibility to integrate three different types of biological data: numerical data, images, and networks which are increasingly available in online resources, therefore being widely applicable to all fields of biology. In the future the growing availability of multi-domain datasets will require the additional integration of 3D volumes (e.g., from 3D MALDI imaging mass spectrometry), pointing to a possible extension of the presented approach.

## Author Contributions

Astrid Junker and Hendrik Rohn wrote the manuscript, Astrid Junker designed the usecases, Hendrik Rohn implemented the tool, Falk Schreiber supervised the project and gave conceptual advice.

## Conflict of Interest Statement

The authors declare that the research was conducted in the absence of any commercial or financial relationships that could be construed as a potential conflict of interest.

## Supplementary Material

The Supplementary Material for this article can be found online at http://www.frontiersin.org/Plant_Systems_Biology/10.3389/fpls.2012.00252/abstract

Supplementary File S1 **HIVE tutorial (PDF)**.

Supplementary File S2 **Raw data for usecase 1 (ZIP; including homeotic gene-regulatory network in SBGN Activity Flows, HIVE excel template with flower expression data, Electron Microscopic image of the *Arabidopsis* flower and the corresponding segmented image)**.

Supplementary File S3 **Raw data for usecase 2 (ZIP; including AFLB3/LEC1 gene-regulatory network in SBGN Process Descriptions, HIVE excel template with seed expression data, schematic image of the *Arabidopsis* seed stages and the corresponding segmented image, high-resolution image of Figure 4A)**.

## Footnotes

^1^http://www.gimp.org

^2^http://www.adobe.com/de/products/photoshop.html
