# Supplementary material for: Visual Analysis of Transcriptome Data in the Context of Anatomical Structures and Biological Networks
Source: Front Plant Sci. 2012 Nov 15;3:252. doi: 10.3389/fpls.2012.00252 (PMC3498740; doi:10.3389/fpls.2012.00252)
Supplement: Supplementary File S1 — HIVE tutorial (PDF). [file 33720_Rohn_DataSheet1.PDF]

Visual analysis of transcriptome data in the context of anatomical structures and biological network

HIVE Tutorial

Installing HIVE

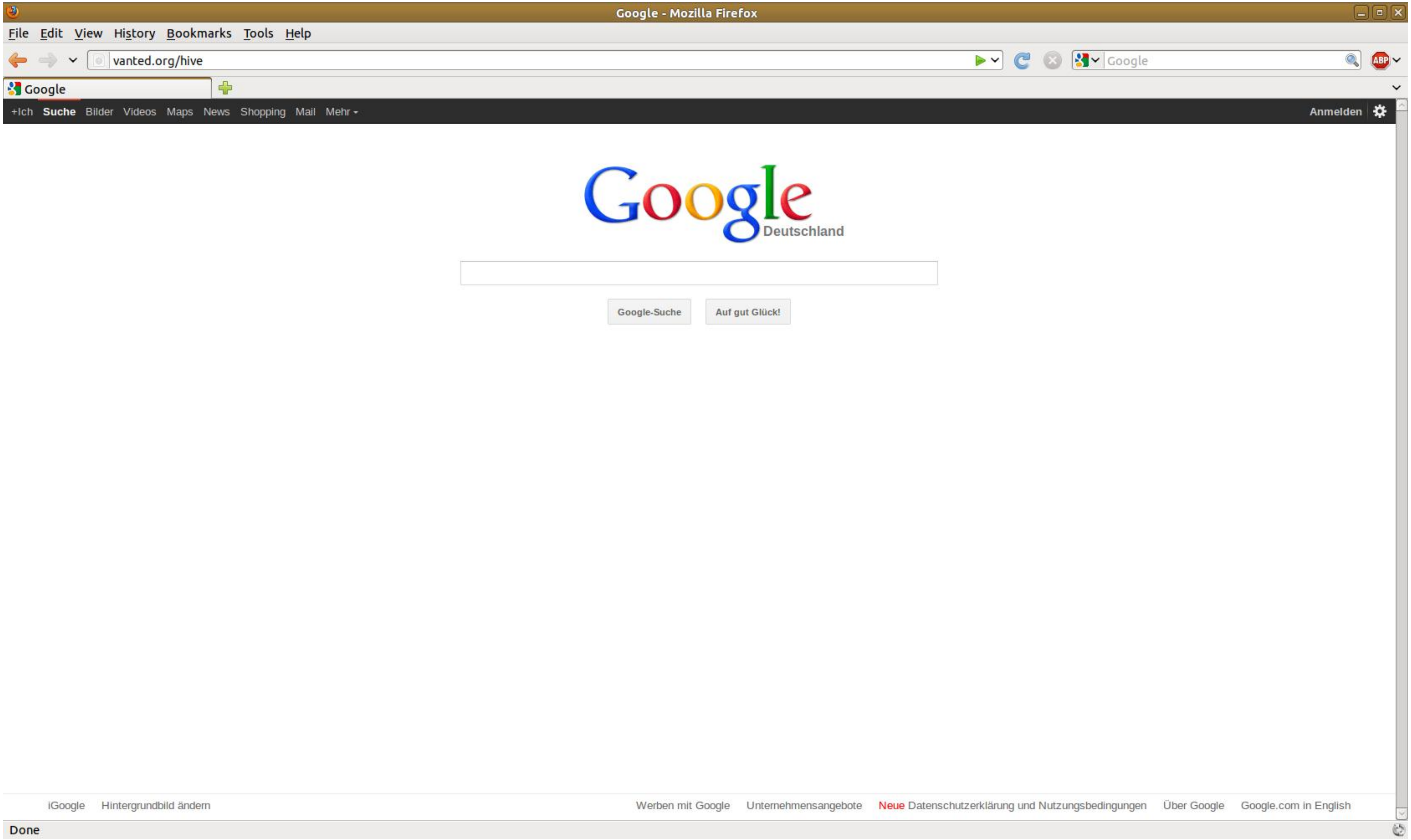

Figure 1 Go to HIVE homepage http://www.vanted.org/hive/

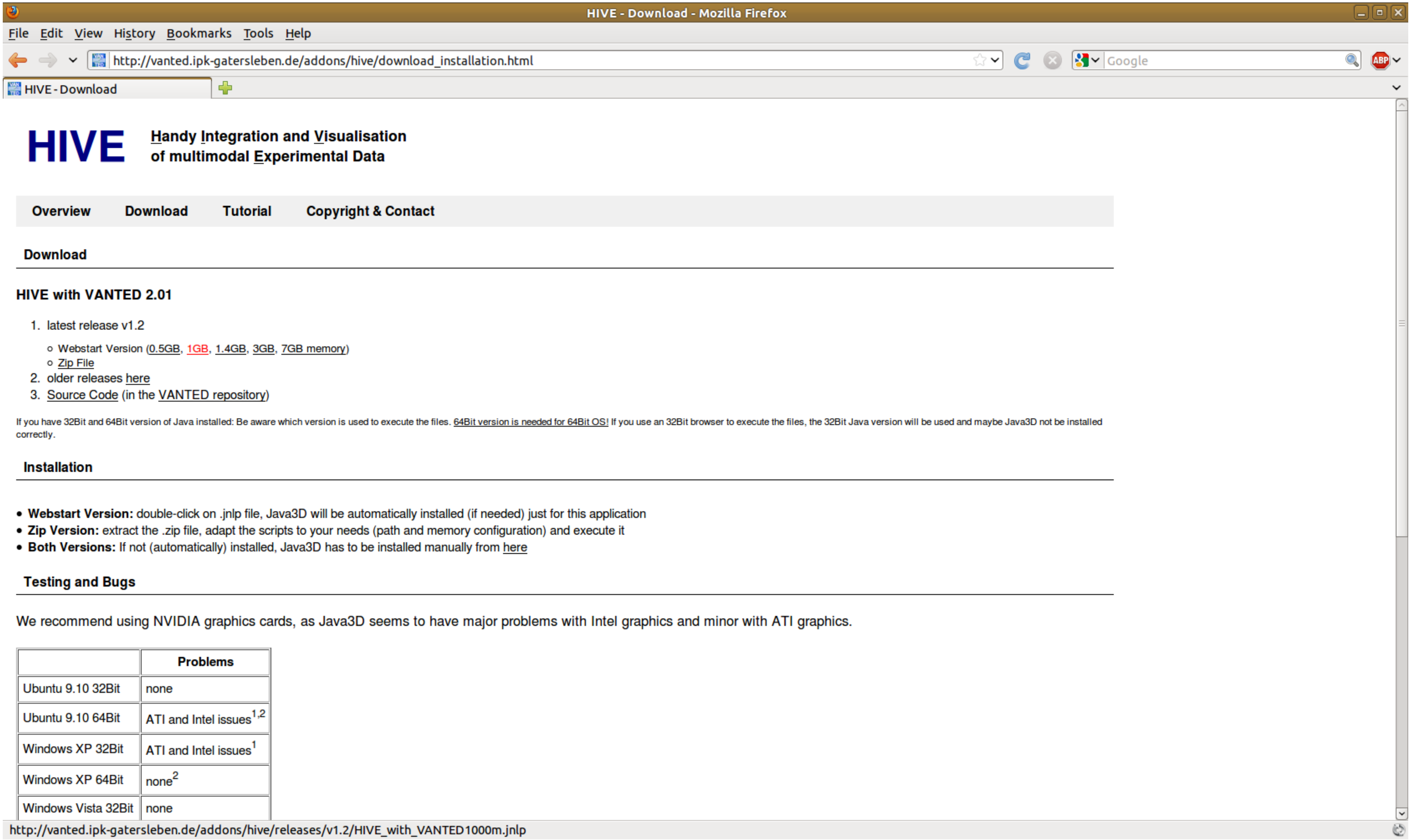

Figure 2 Start 1GB HIVE version by using webstart. For Windows users usually the 32Bit Java version is installed, therefor not more than 1.4GB are supported. If you need more memory, please install a 64Bit Java version and start the webstart file with 64Bit Java.

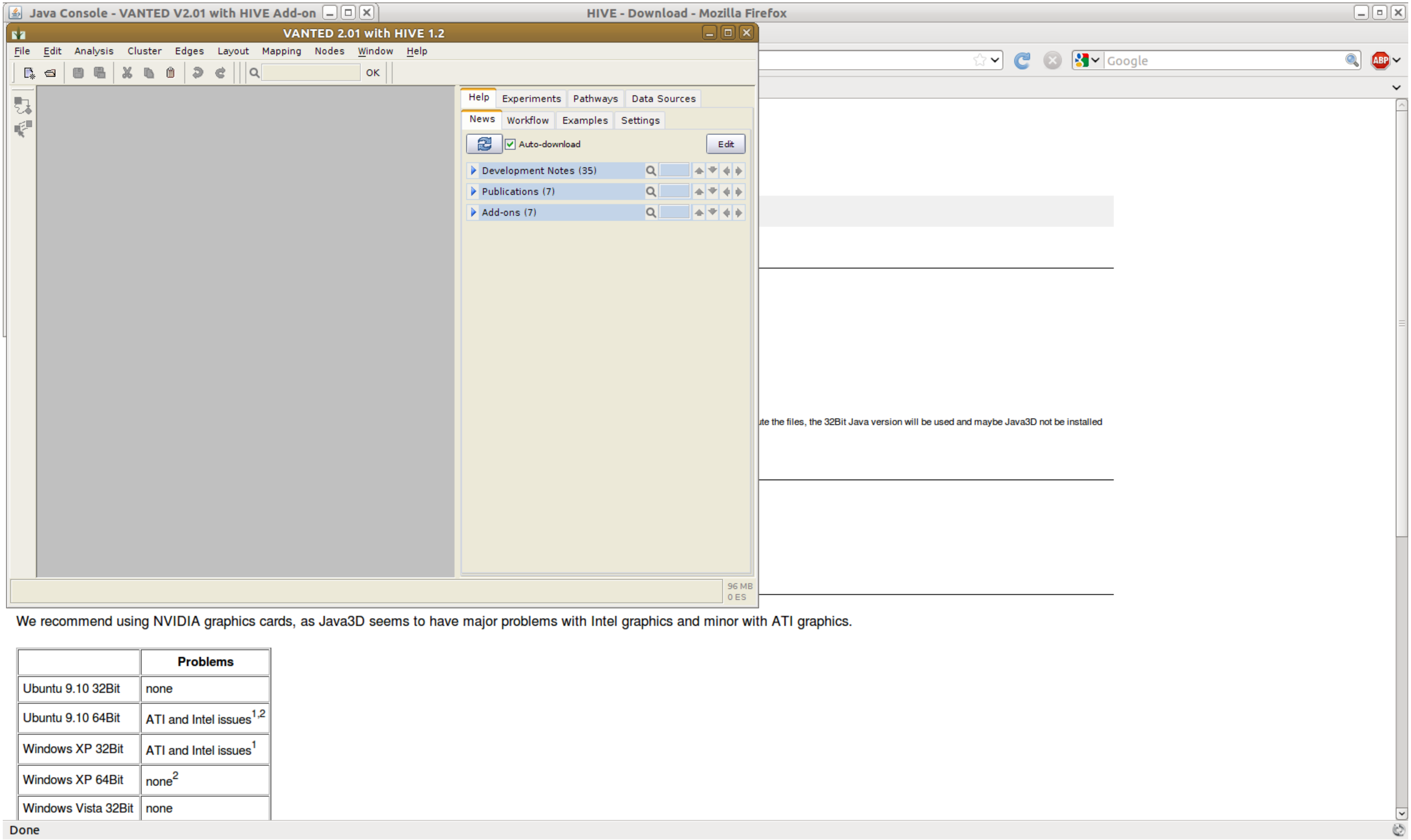

Figure 3 Please wait until HIVE was downloaded and installed correctly. Webstart checks on each startup for new versions.

Importing data into HIVE

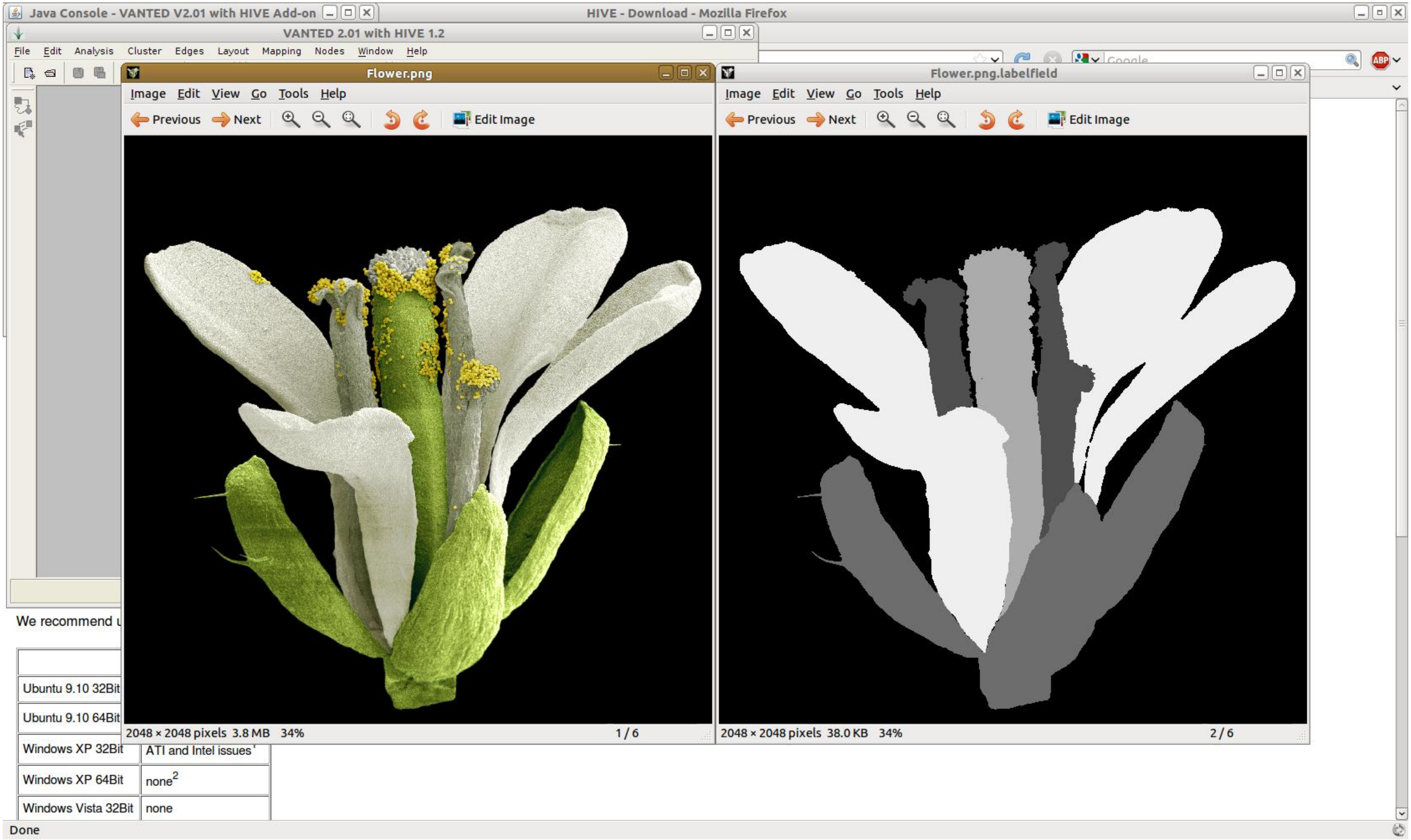

Figure 4 Take an image and generate a labelfield by image segmentation. This procedure assigns each pixel to an anatomical structure (organ, tissue, ...). The labelfield image is a gray-scale image with the same name as the original image and ".labelfield" appended. See Figures 32 – 35 for useful hints for manual image segmentation.

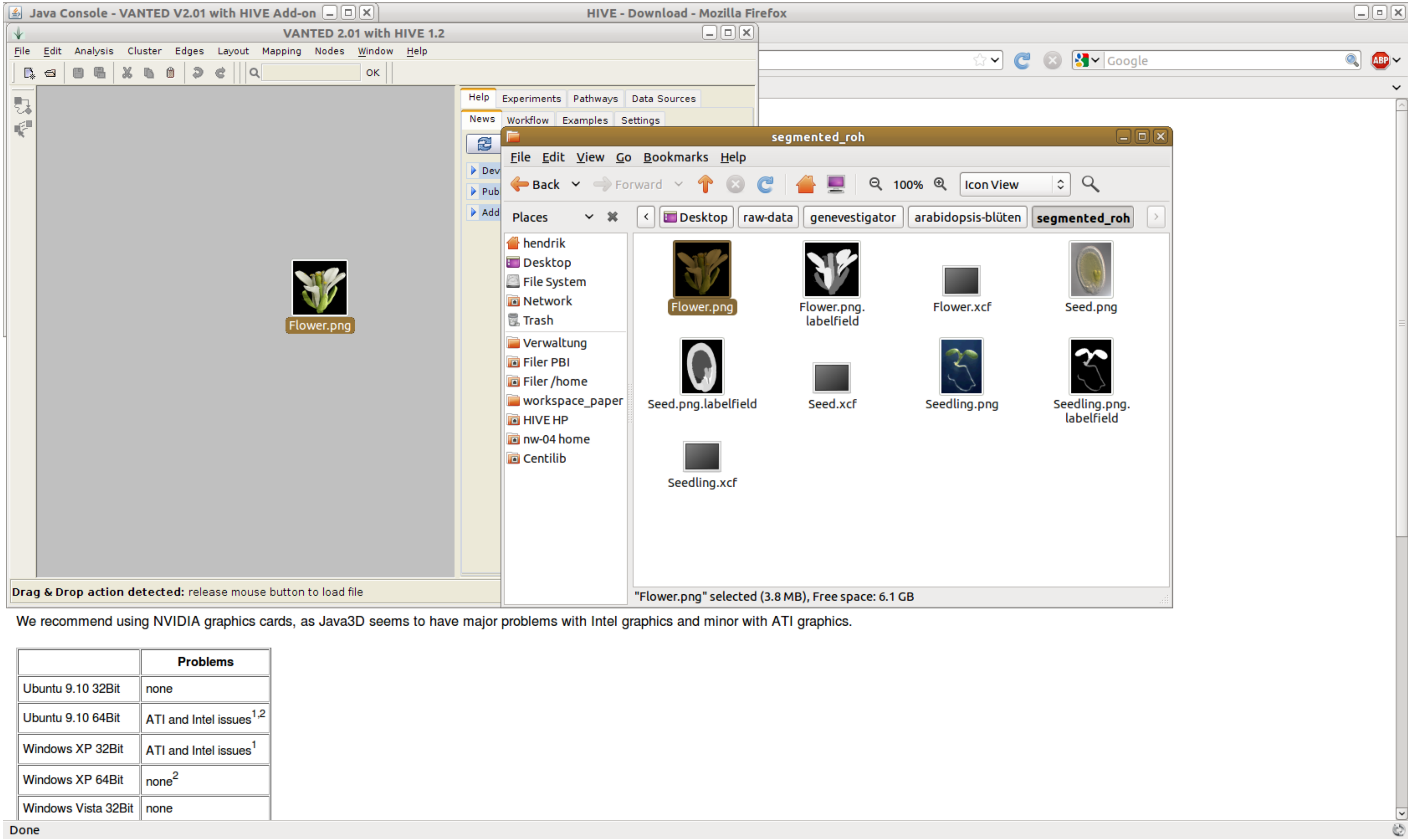

Figure 5 Import the original image by Drag and Drop. Please note, that the labelfield image has to be in the same directory and will be automatically imported together with the original image.

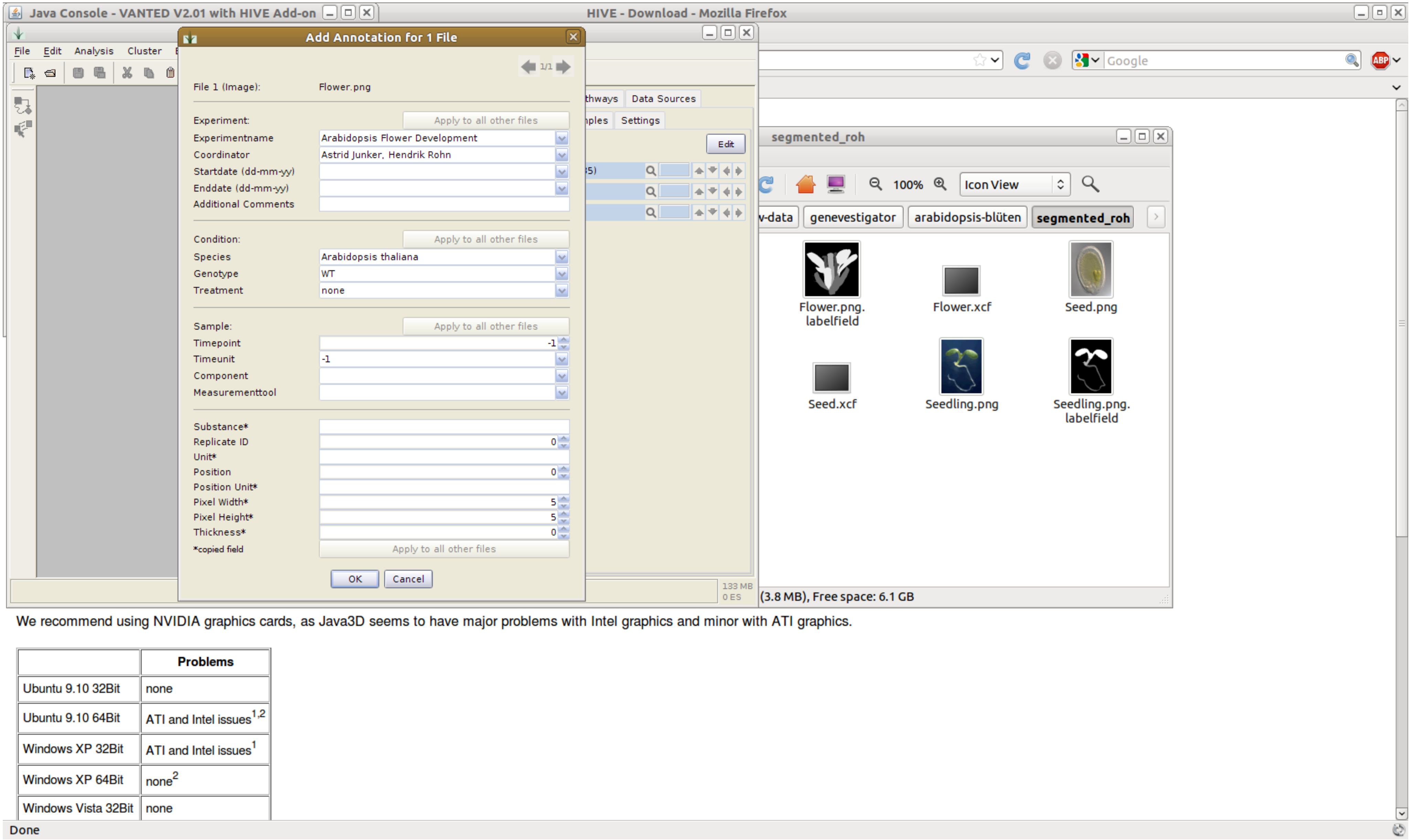

Figure 6 Specify the image metadata in the following dialog, which later can be used for search and exploration tasks.

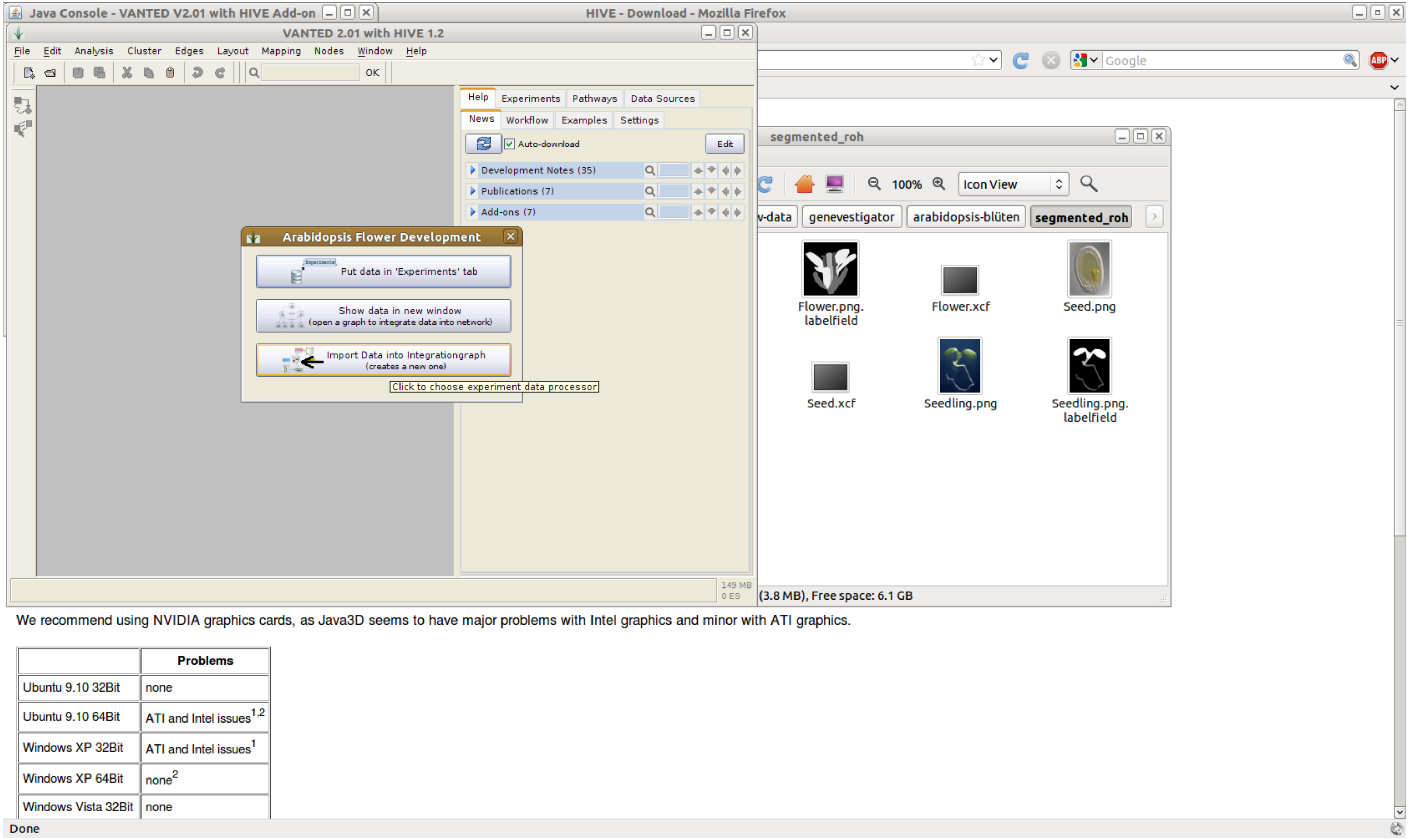

Figure 7 Choose to directly import this dataset (which consists of only one image) into a new integrationgraph.

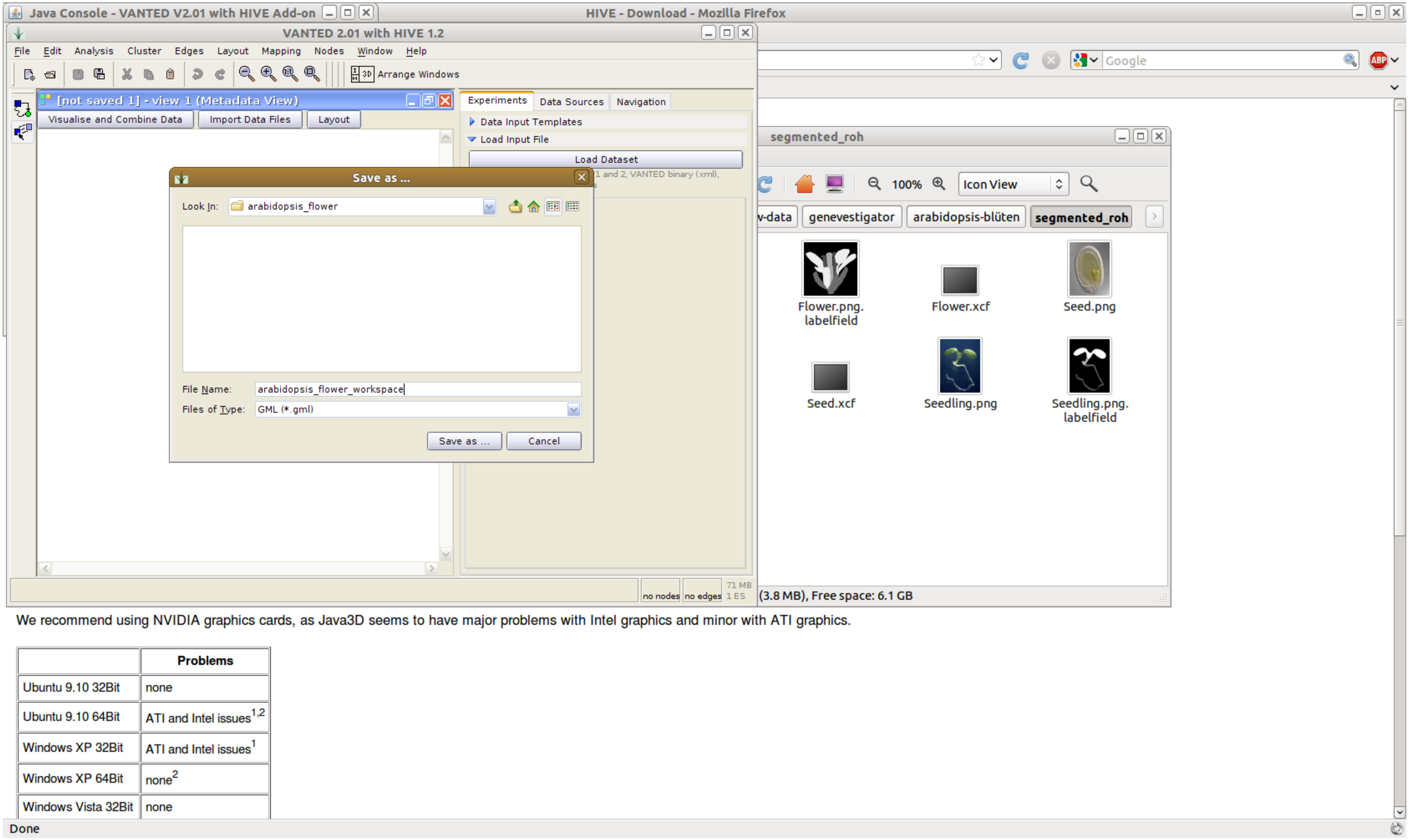

Figure 8 A project directory has to be specified. Please create a new directory and save the integrationgraph into this directory. Please note, that all actions performed in the subsequent steps are storing and loading data from this project directory. You may send the directory (usually zip'ed) to collaborators, which can be restored by HIVE in order to continue to work with the project.

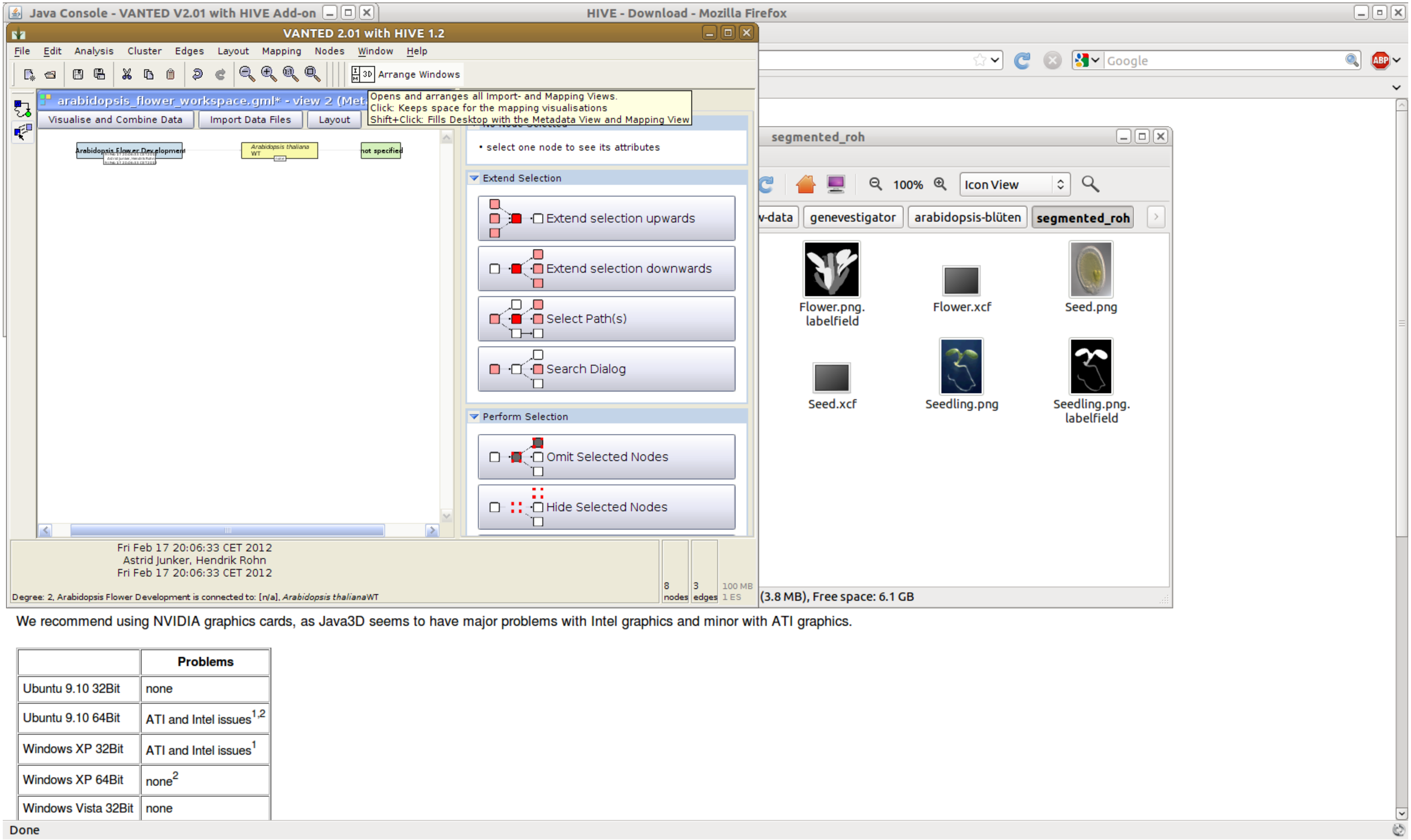

Figure 9 The integrationgraph is created and the dataset imported. Use the "Arrange Windows" button at the top to properly layout the HIVE frame. Please note, that it is advised to have a high screen resolution to be able to productively work with HIVE. A resolution of at least 1600x1050 pixels is advised.

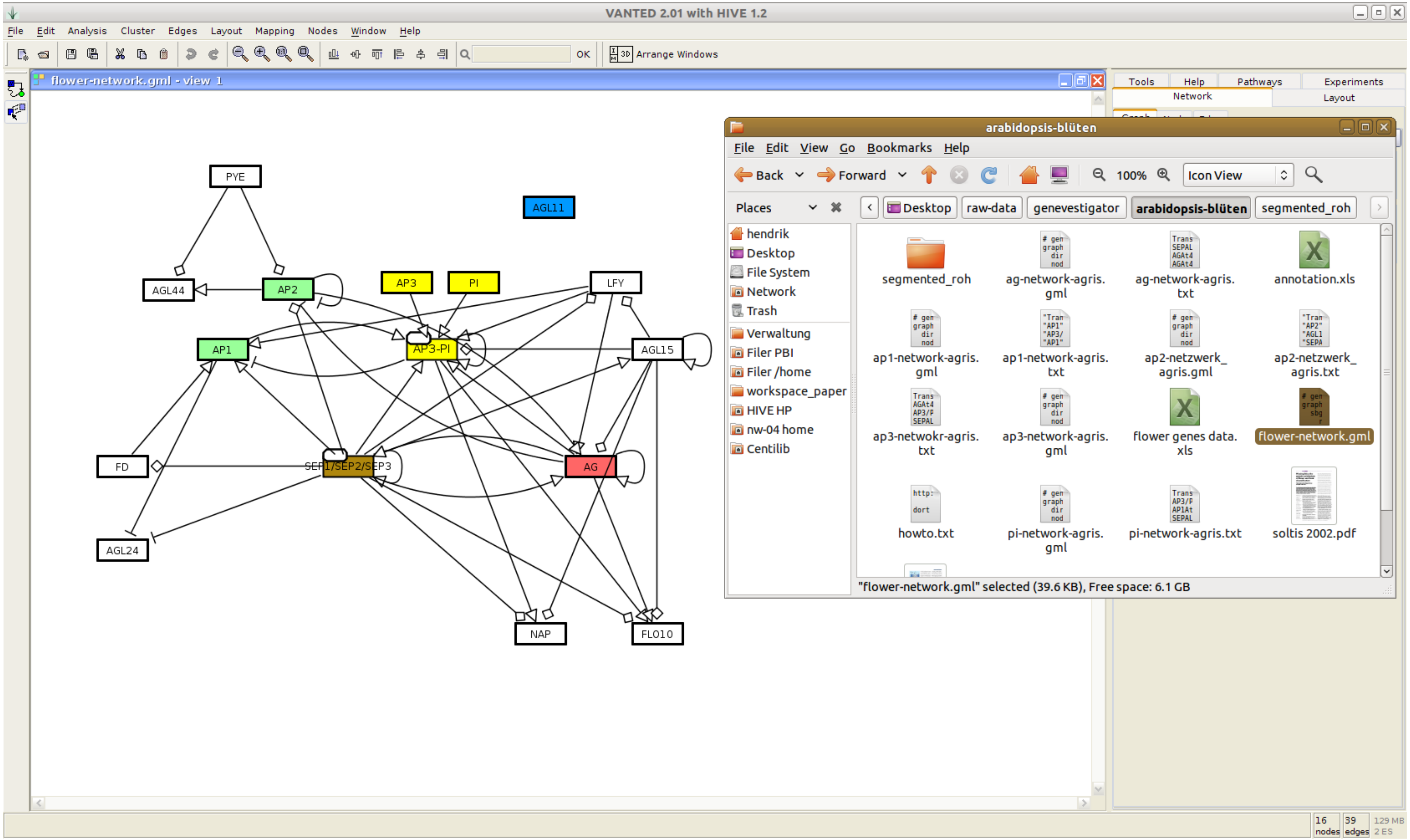

Figure 10 Choose a network file from your file-system. For visual impression the network is shown here, but this is not part of the tutorial. Please note that the network import step is optional.

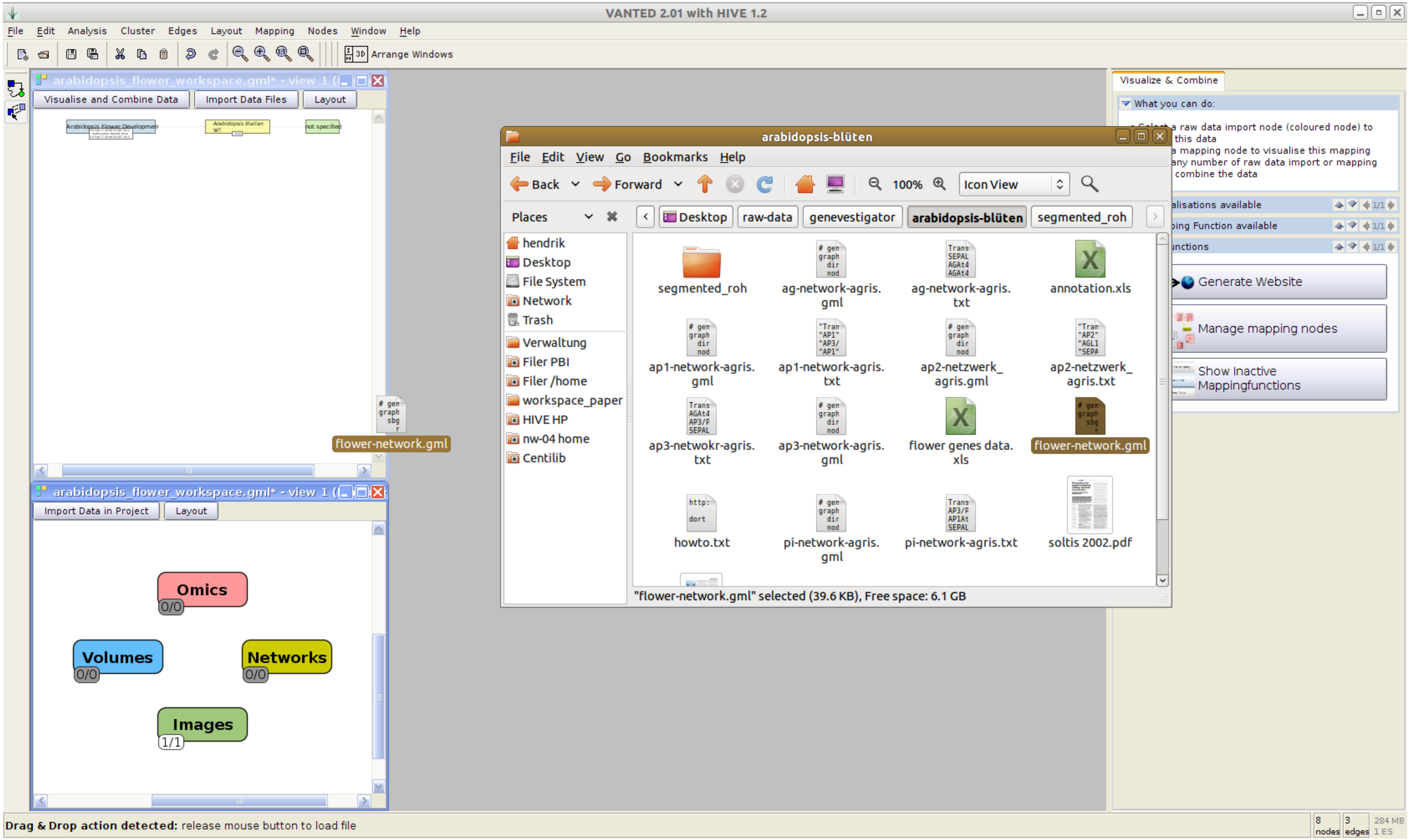

Figure 11 Again Drag and Drop the file onto the HIVE frame.

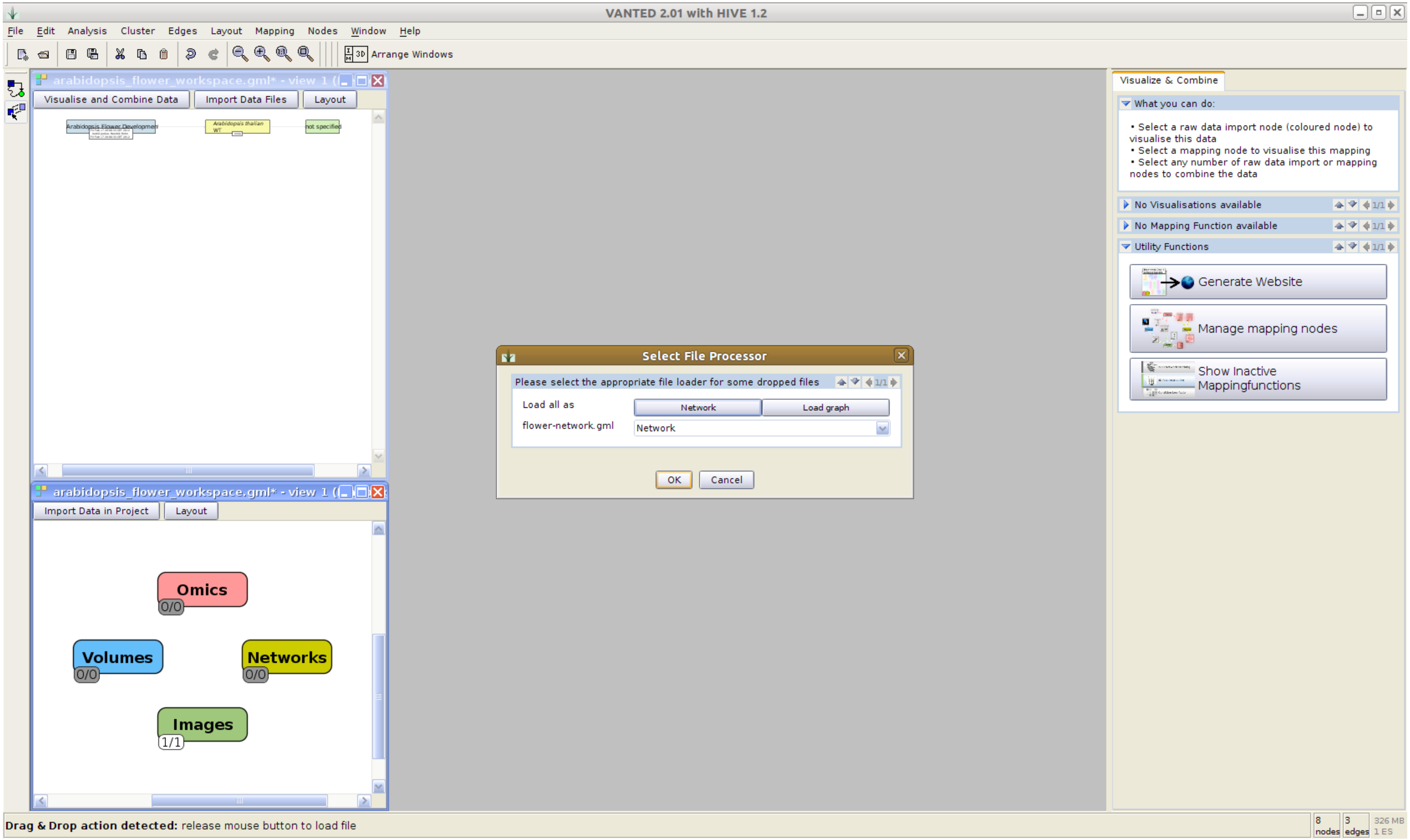

Figure 12 You may now choose to directly load the network as a graph, or interpret it as an dataset consisting of one network. Choose latter option.

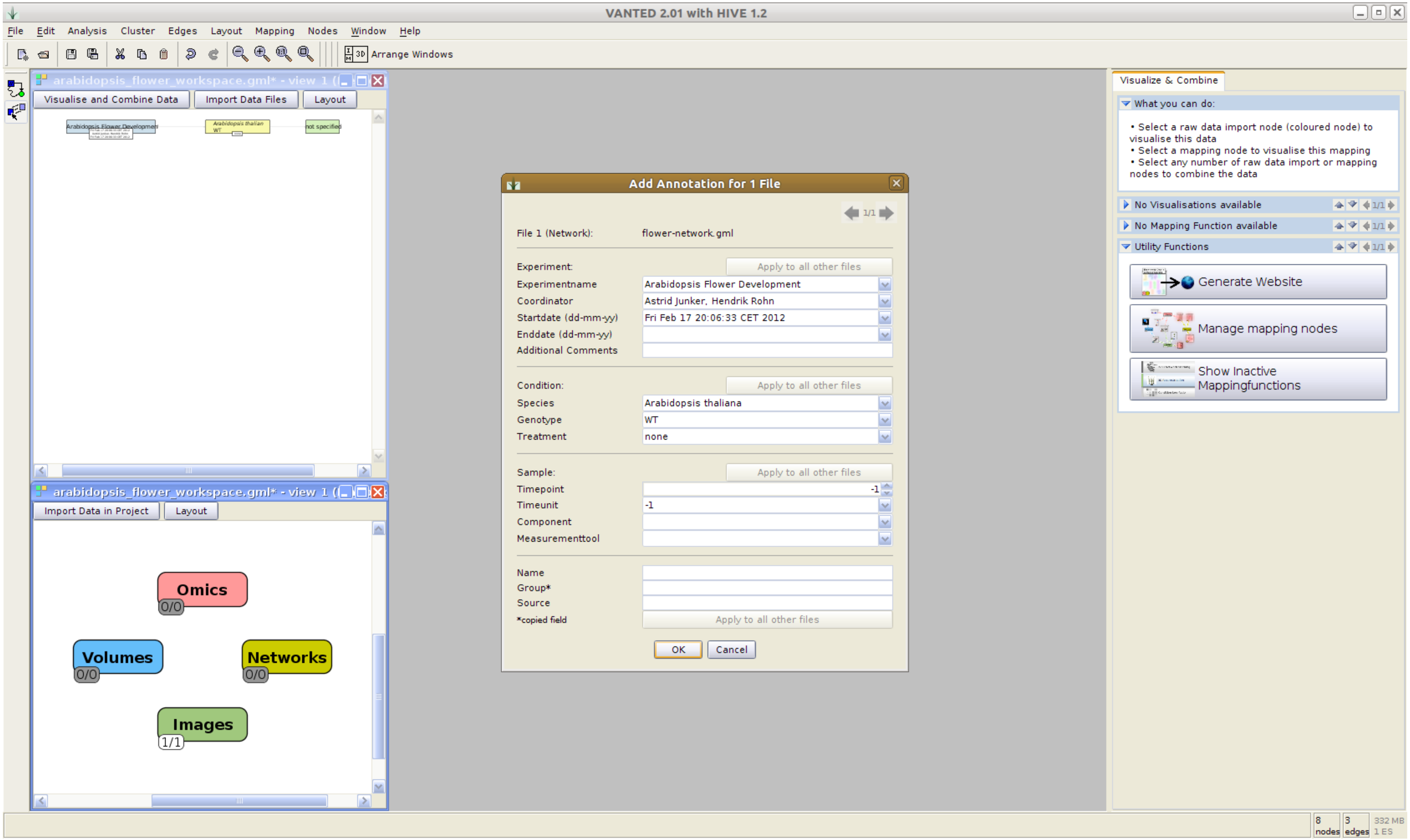

Figure 13 Again specify the metadata. After clicking on "OK" the network shall be imported into the integrationgraph. Note that both views at the left (MetadataView, top and MappingGraph, bottom) get updated by newly imported data.

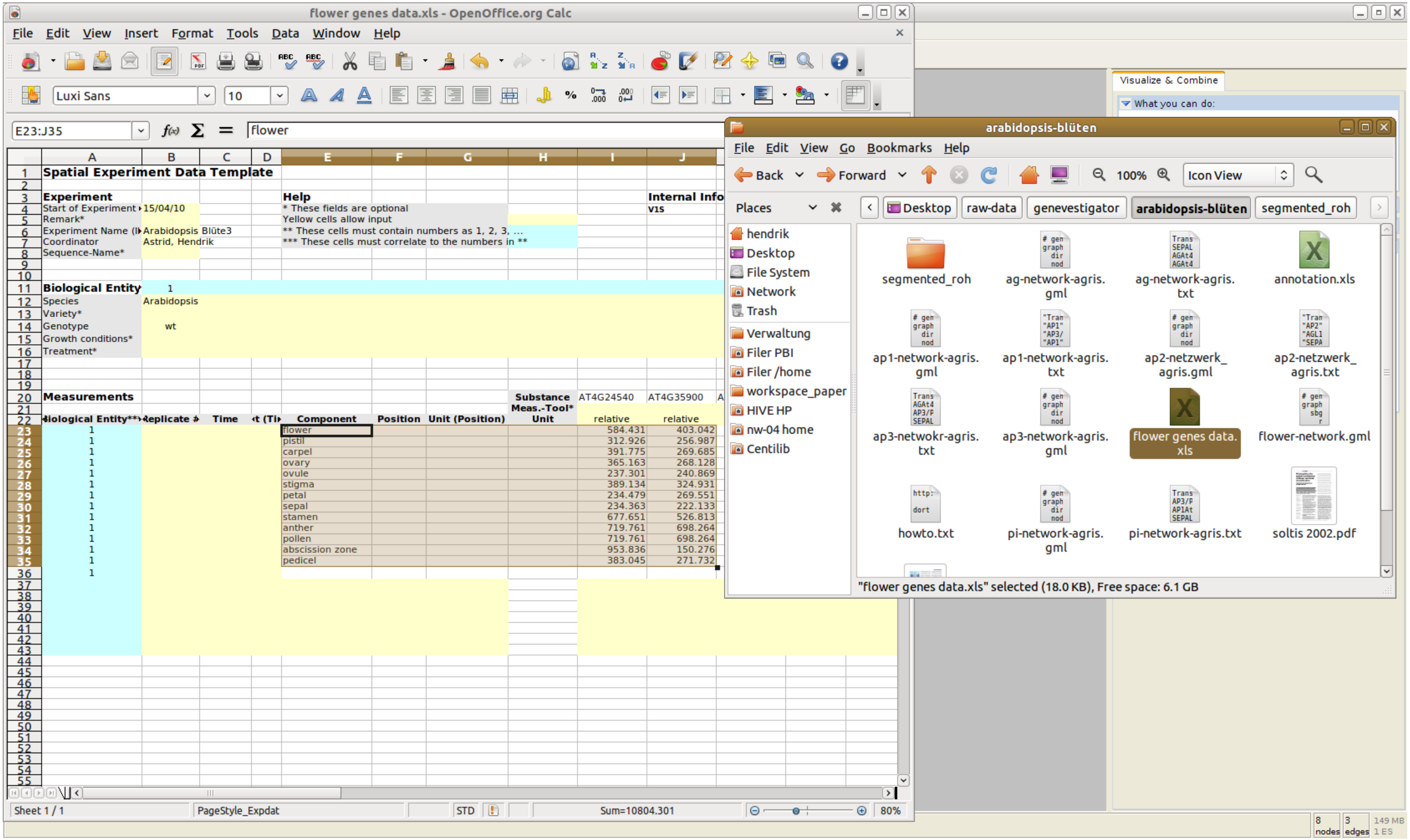

Figure 14 Choose your experimental data set. A template can be found in the tab "Experiments" → "Data Input Templates" by clicking on "Spatial Experiment Data" and saving it to your filesystem. The file will automatically be opened by your default Excel application and can be filled as seen in the left.

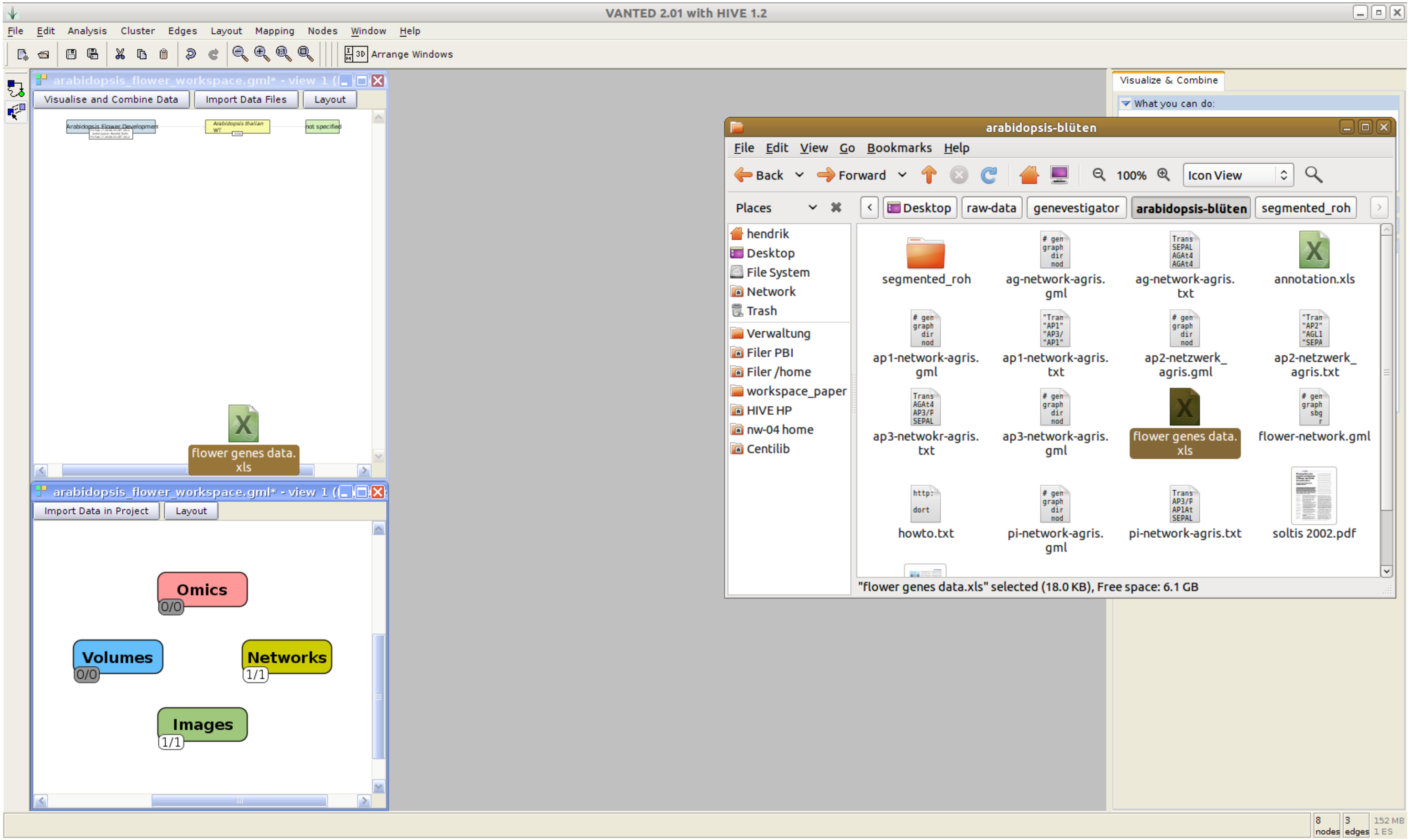

Figure 15 Again Drag and Drop the file onto the HIVE frame and import the dataset into the integrationgraph. In order to isolate context-specific datasets during import, choose the dialog option "Put data in Experiments tab" during the data import. Otherwise choose "Import Data into integrationgraph".

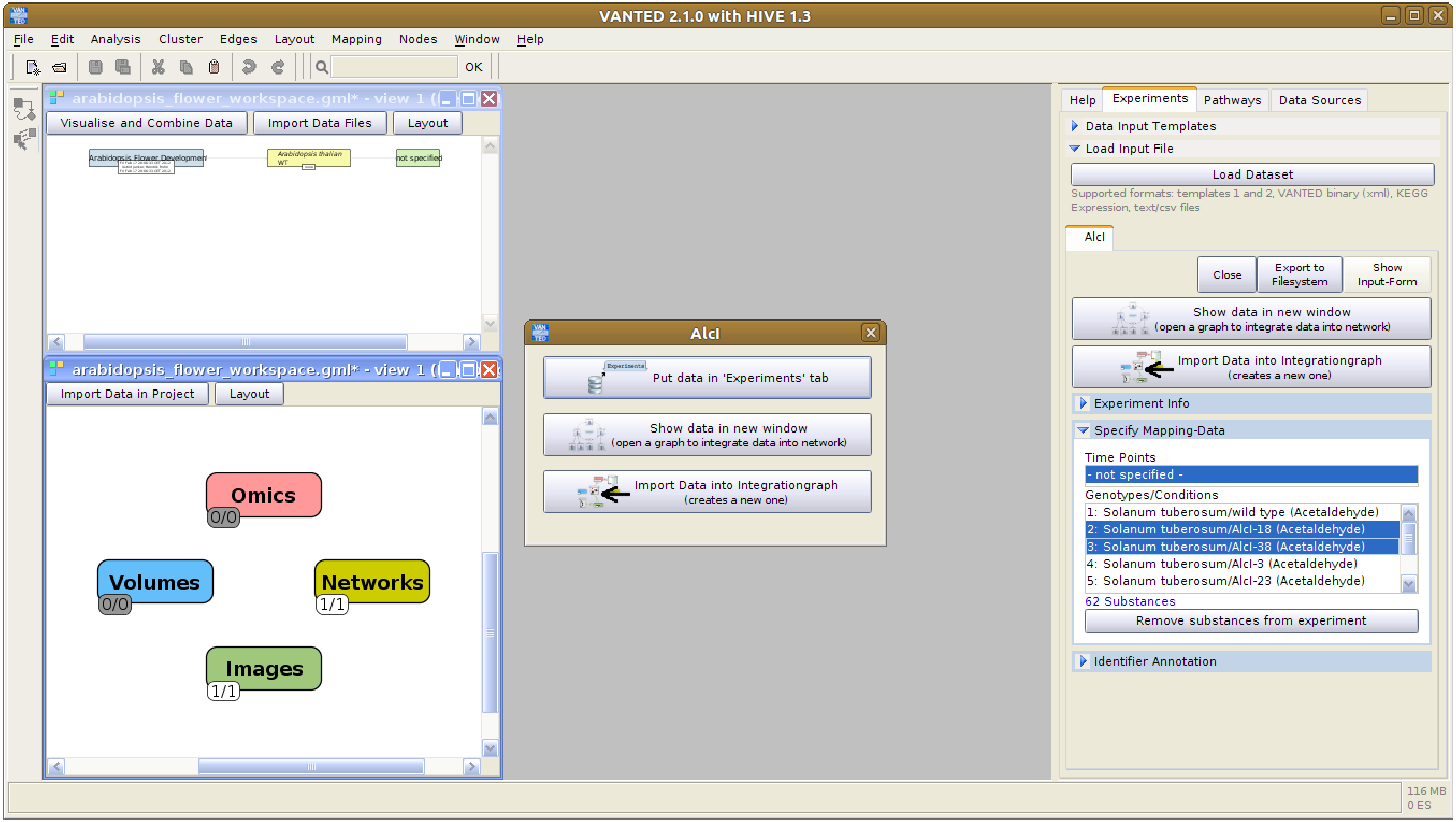

Figure 16 For isolating context-specific datasets, the data will appear in the tab on the right. Here you can remove substances from the loaded dataset (button "Remove substances from experiment") and choose the conditions and timepoints of interest as well (select entries in folder "Specify Mapping-Data"). Finally, press button "Import into integrationgraph" to import the selected context-specific dataset into HIVE.

Map data

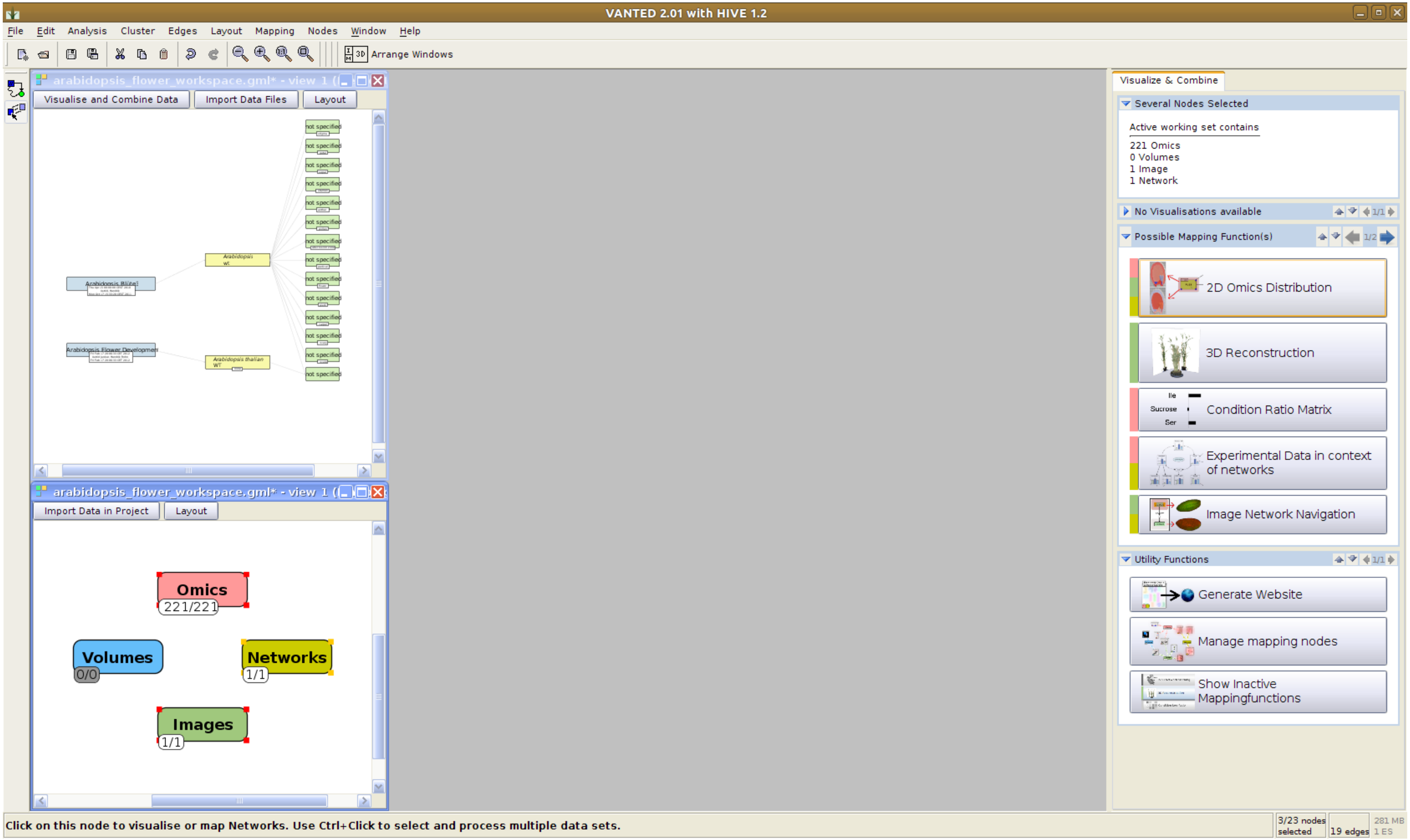

Figure 17 Finally all your data is imported. Select the "Omics", "Networks" and "Images" nodes in the MappingGraph and select the mapping function "2D Omics Distribution" in the tab at the right side.

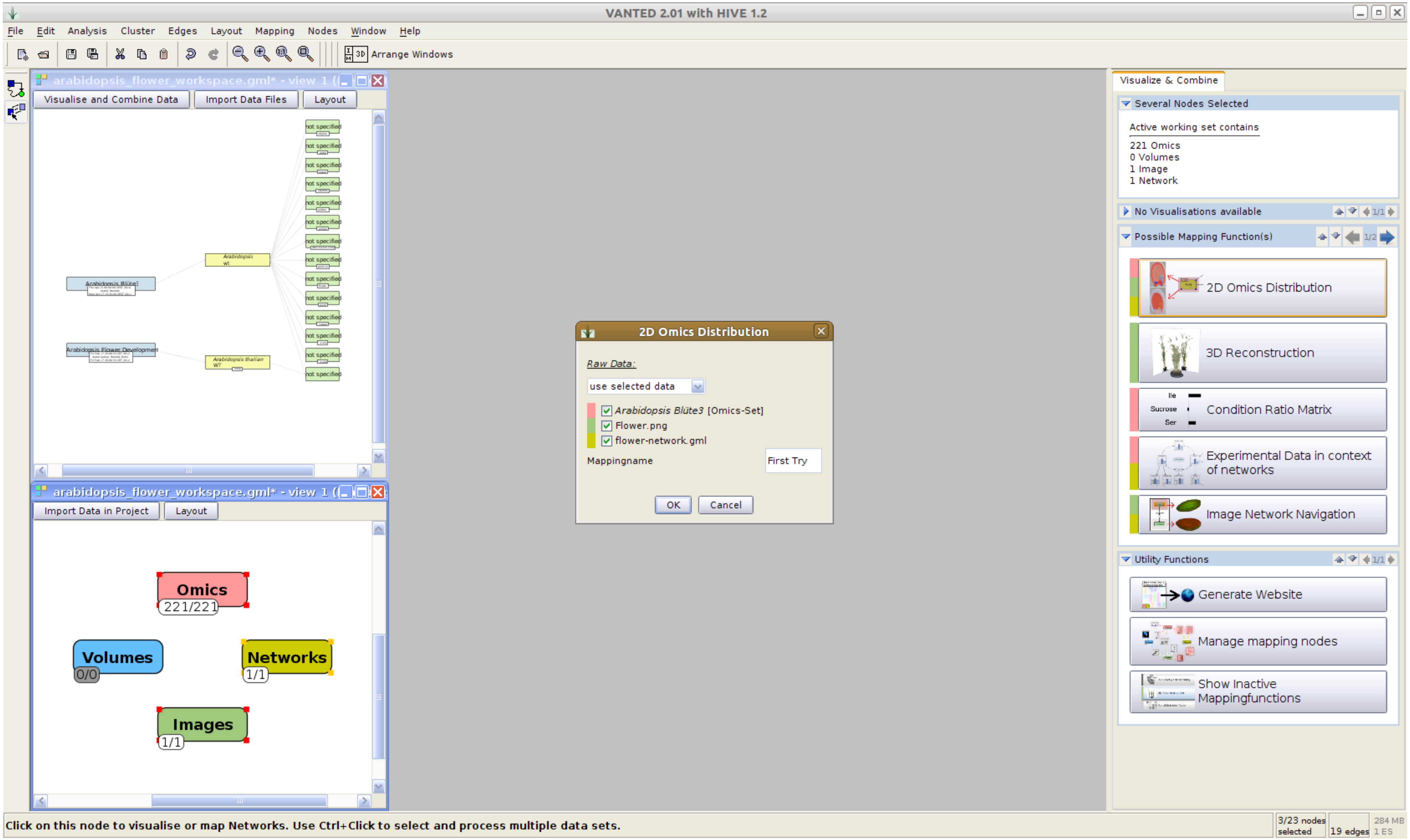

Figure 18 Choose the datasets to be part of the mapping. Here we will need all datasets. Please note that it is possible to perform the mapping without a network in order to generate only colour-coded images.

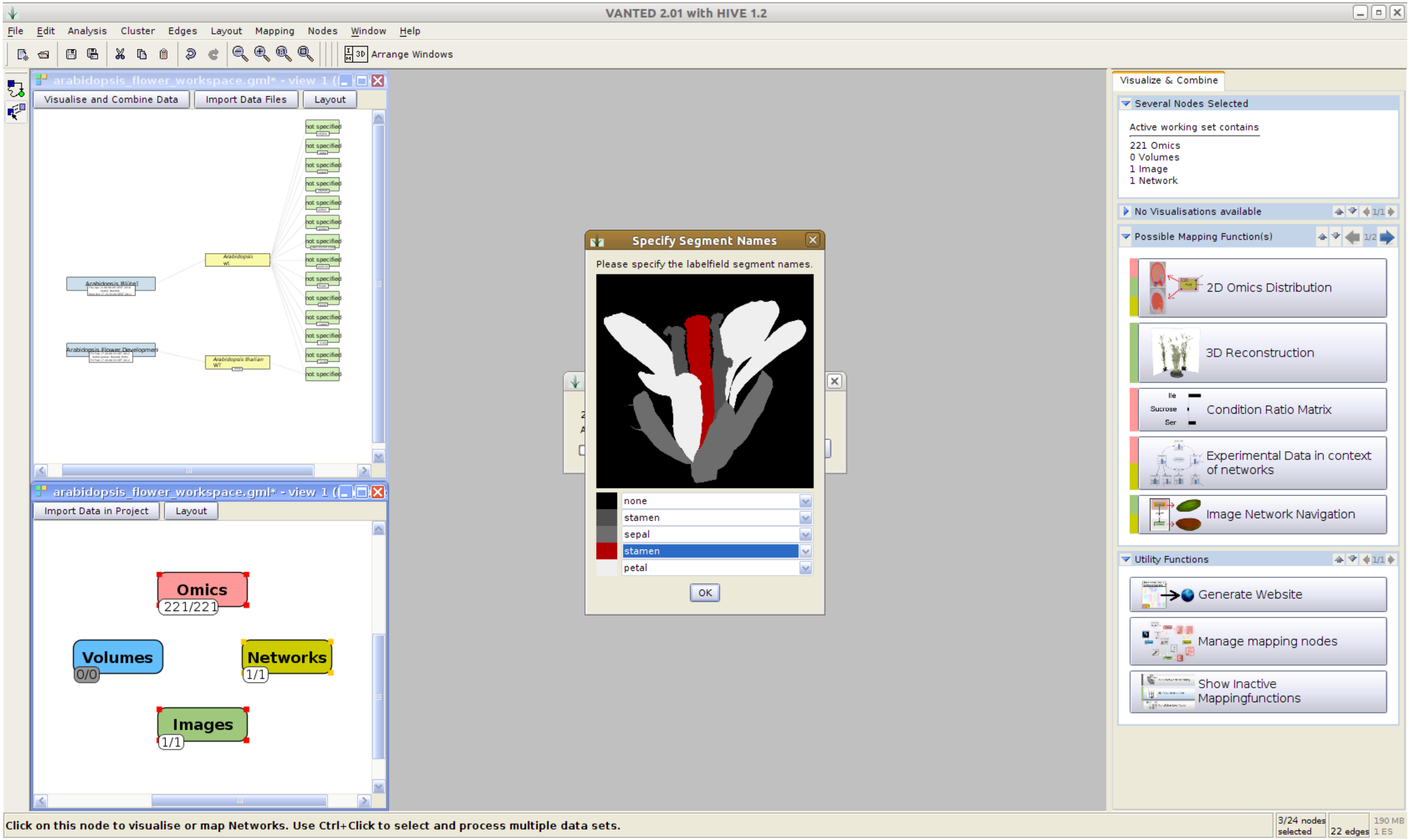

Figure 19 Link the organ names (field "Component") of the experimental dataset to the gray shades of the labelfield image.

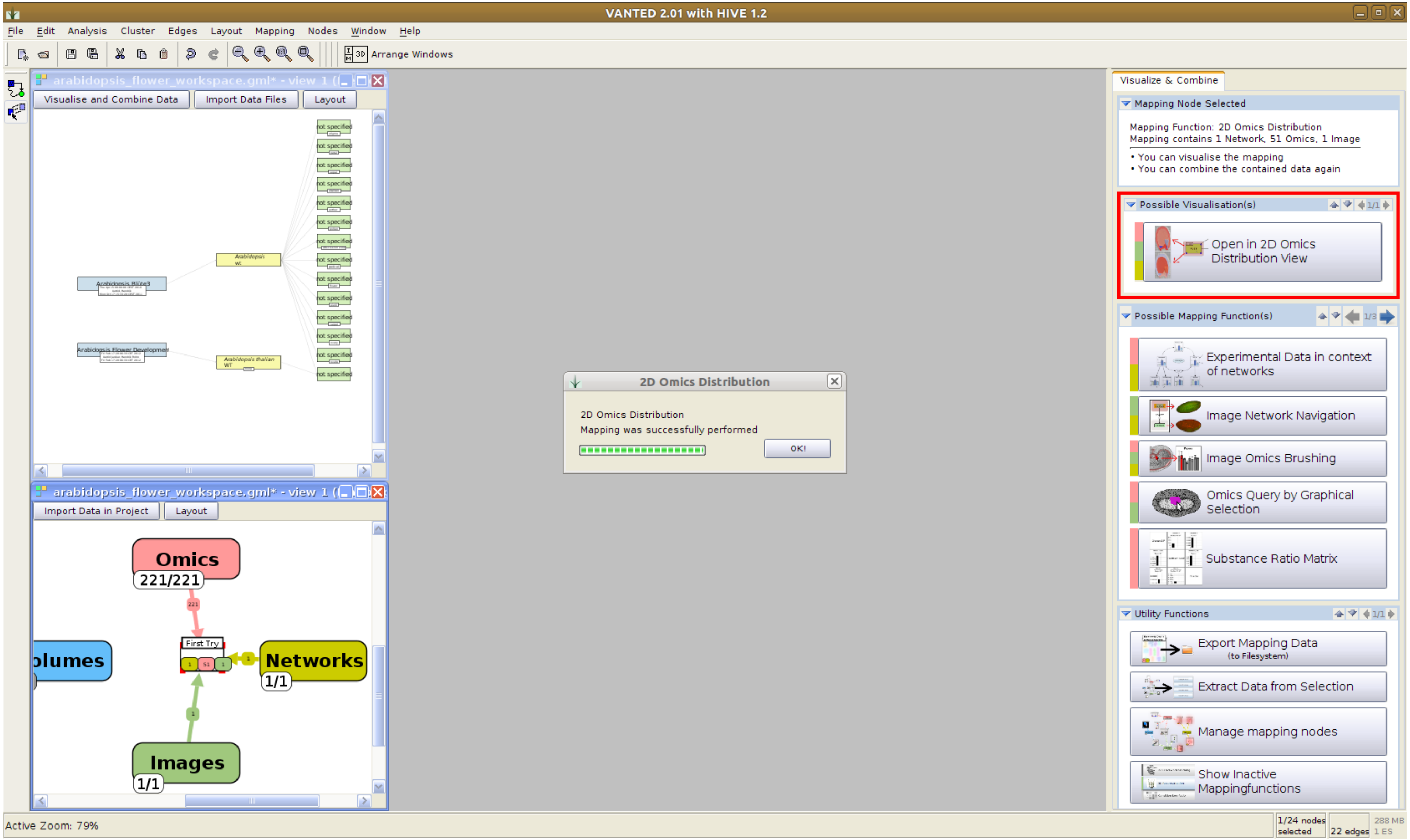

Figure 20 Wait until the mapping is performed, select the newly created mapping node (if not already selected) and choose the visualization "2D Omics Distribution View" by clicking at the respective button in the right tab.

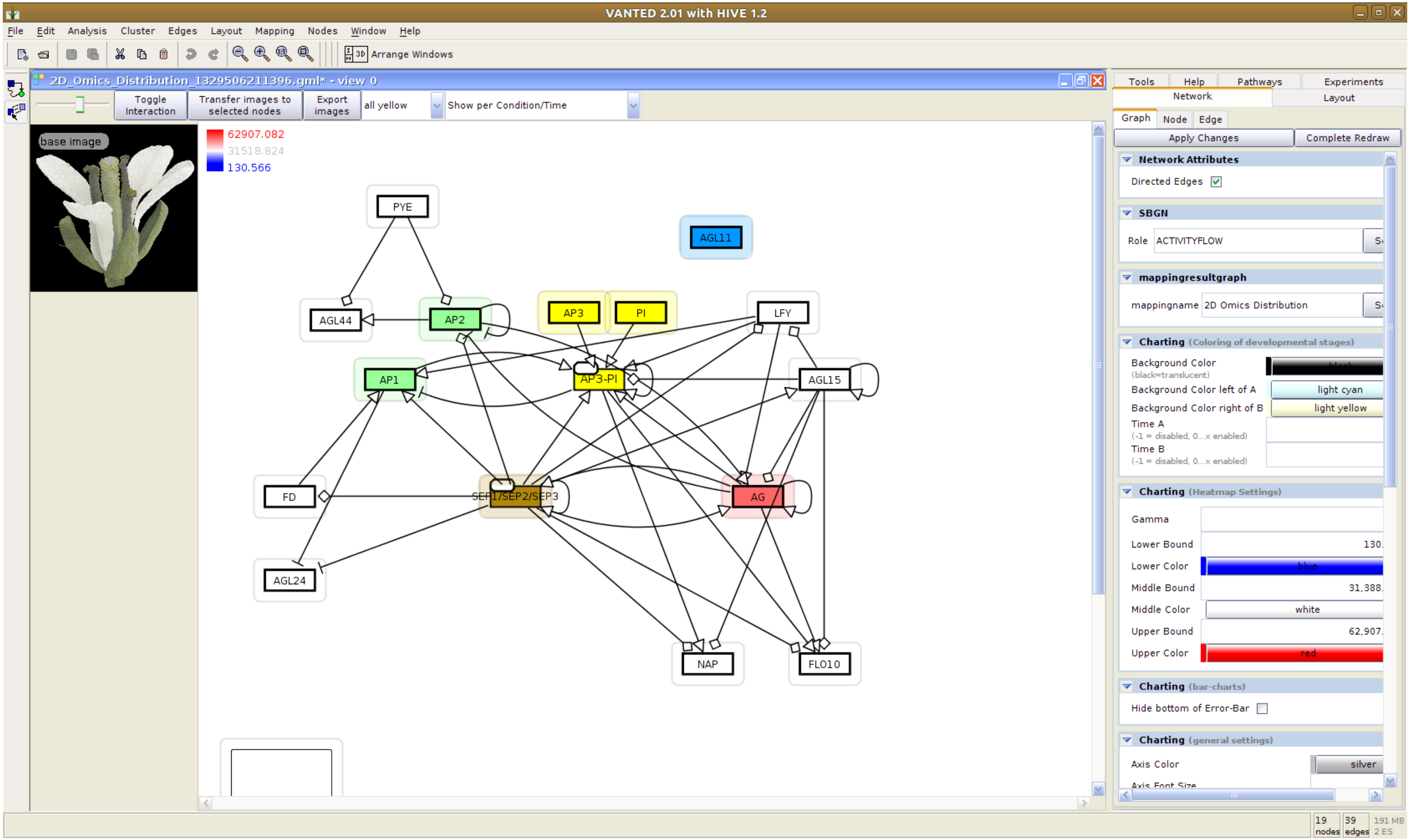

Figure 21 The mapping is successful. All nodes with transparent shades contain mapped data.

Generate colourcoded images

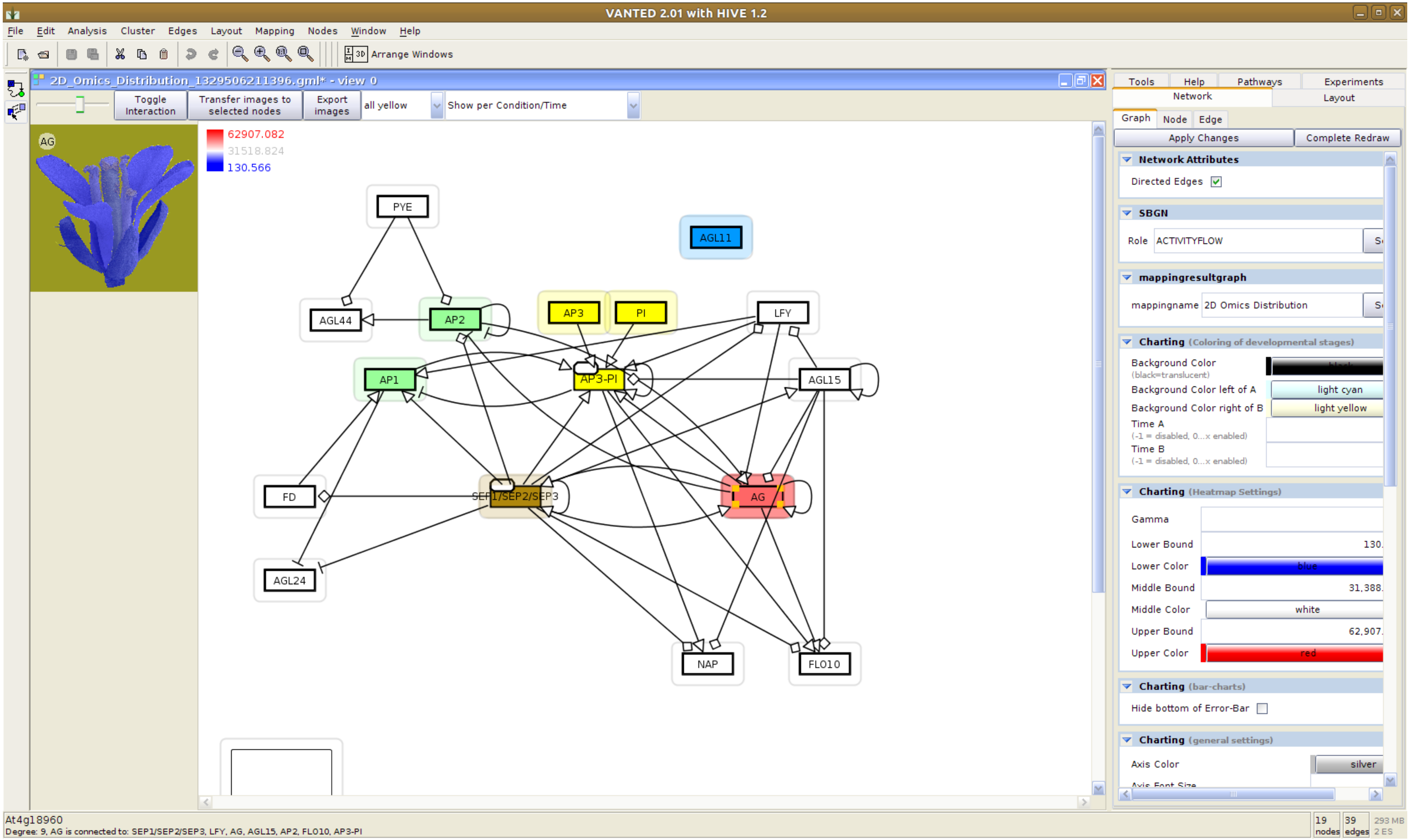

Figure 22 Move the mouse pointer over such a node to generate the colourcoded image in the image area (left side). You can enlarge the image area by moving the mouse pointer over it and scrolling your mouse wheel.

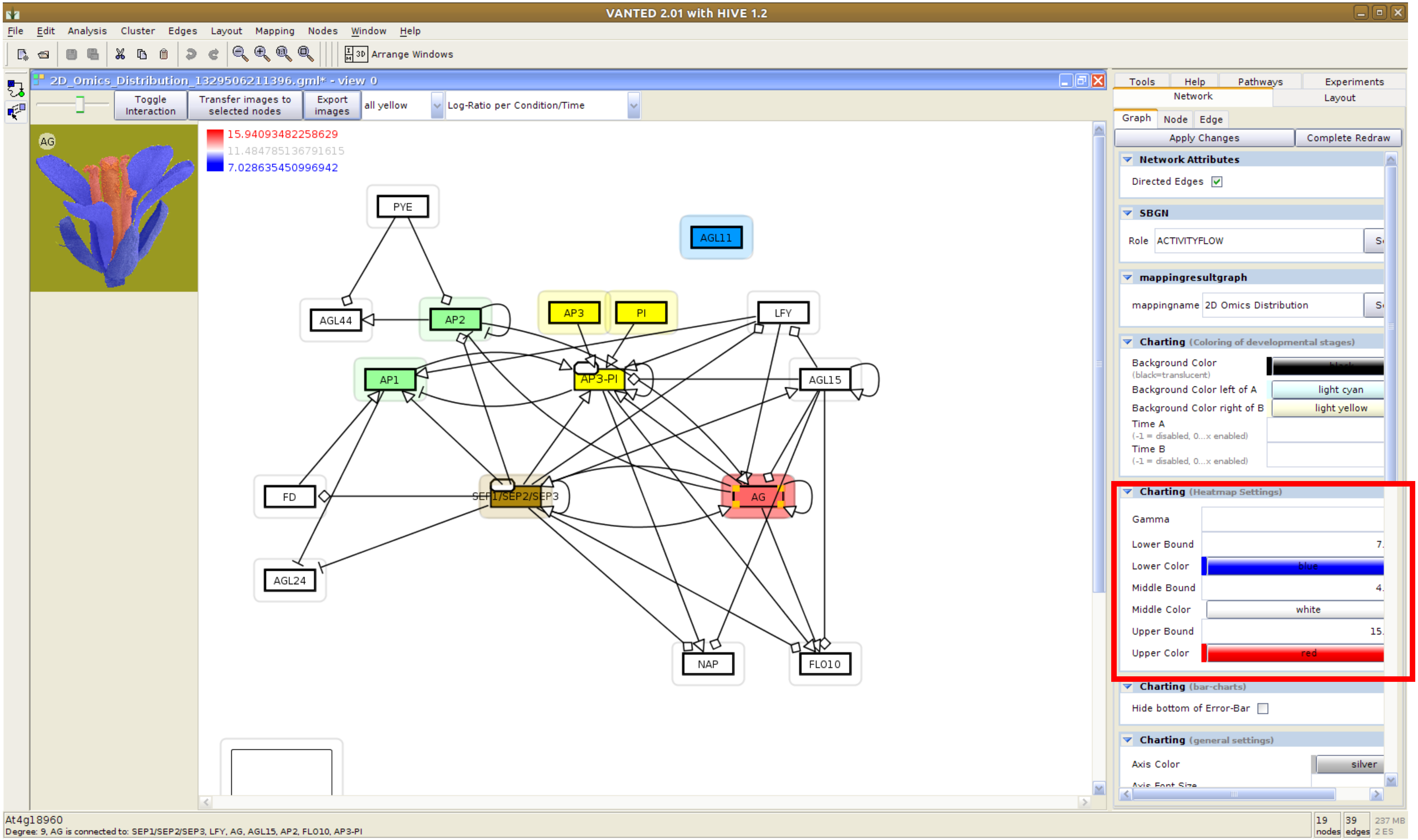

Figure 23 For expression data it is useful to apply a logarithmic scale. Please choose the "Log-Ratio per Condition/Time" option from the combo box at the top. The image and the colourmap will be automatically updated. In order to manually change the colourmap adapt the settings in the right hand panel and press "Apply Changes".

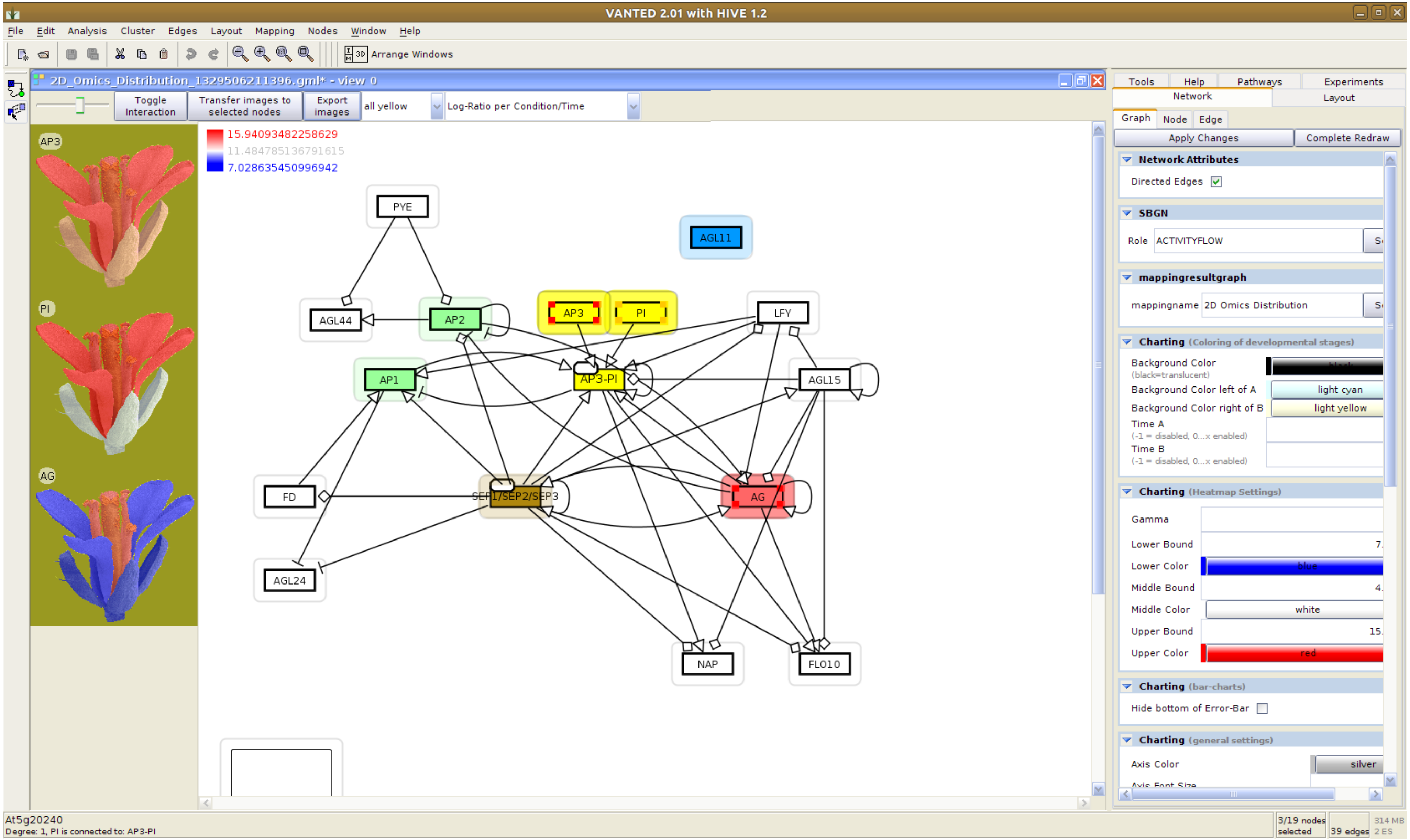

Figure 24 In order to compare transcription patterns, several nodes can be selected. Either draw a frame around the nodes or click on each node while pressing <Ctrl> key. The imageview gets updated by adding one image for each node.

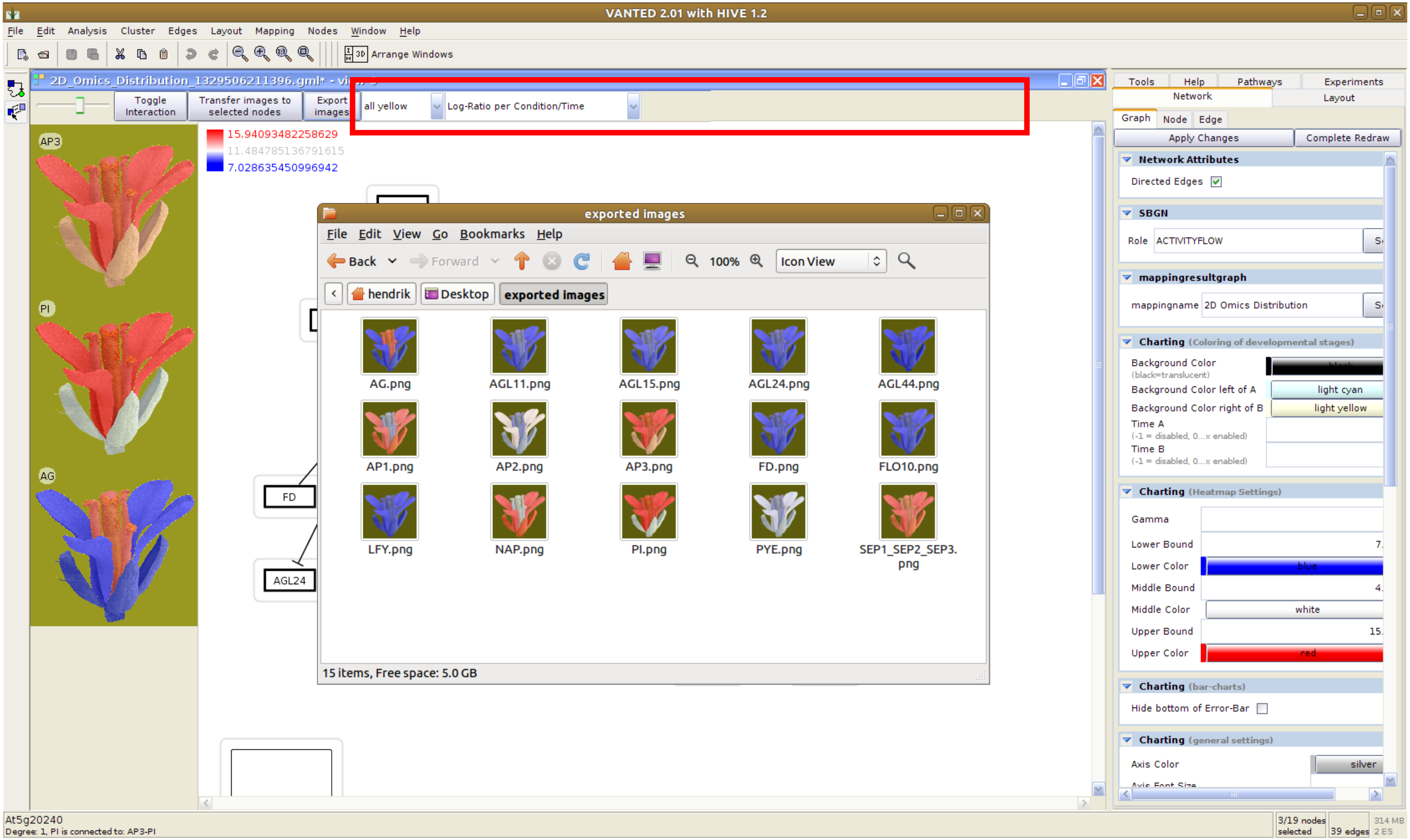

Figure 25 In order to export all images out of the imageview to the filesystem, press button "Export images". The exported images represent the "Integration of transcriptomics data and 2D images" step. For datasets with multiple conditions/timepoints the user may use the combobox in order to switch between conditions and timepoints (see next Figure).

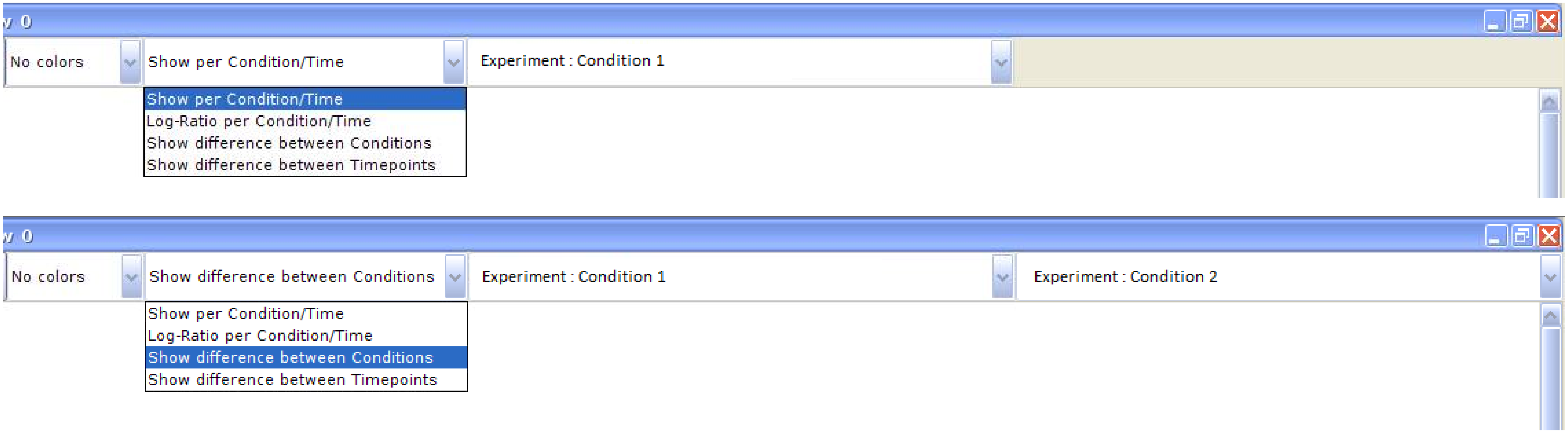

Figure 26 using the combobox the user may interactively switch between the visualized condition/timepoint in an absolute ("Show per Condition/Time") or logarithmic ("Log-Ratio per Condition/Time") manner. Furthermore, two conditions can be compared ("Show difference between Conditions"). This option will calculate the absolute difference between the expression value of condition 1 compared to condition 2 for each segment. These values are again visualized by colourcoding the anatomical structure. Please note that the colourmap will be adapted to the generated values. Two timepoints can be compared analogously.

The combobox at the left ("No colors") enables users to set the colour for segments with missing data. If data is missing in the template, an internal error occurred or the user explicitly assigned part of the image as background (segment "none"), a specific value-independent colouring will occur: The user may choose to 1) just use the labelfield image, 2) gray-fade the area, 3) gray-stripe the area, 4) yellow-fade the area or 5) yellow-stripe the area.

Integrate images into networks

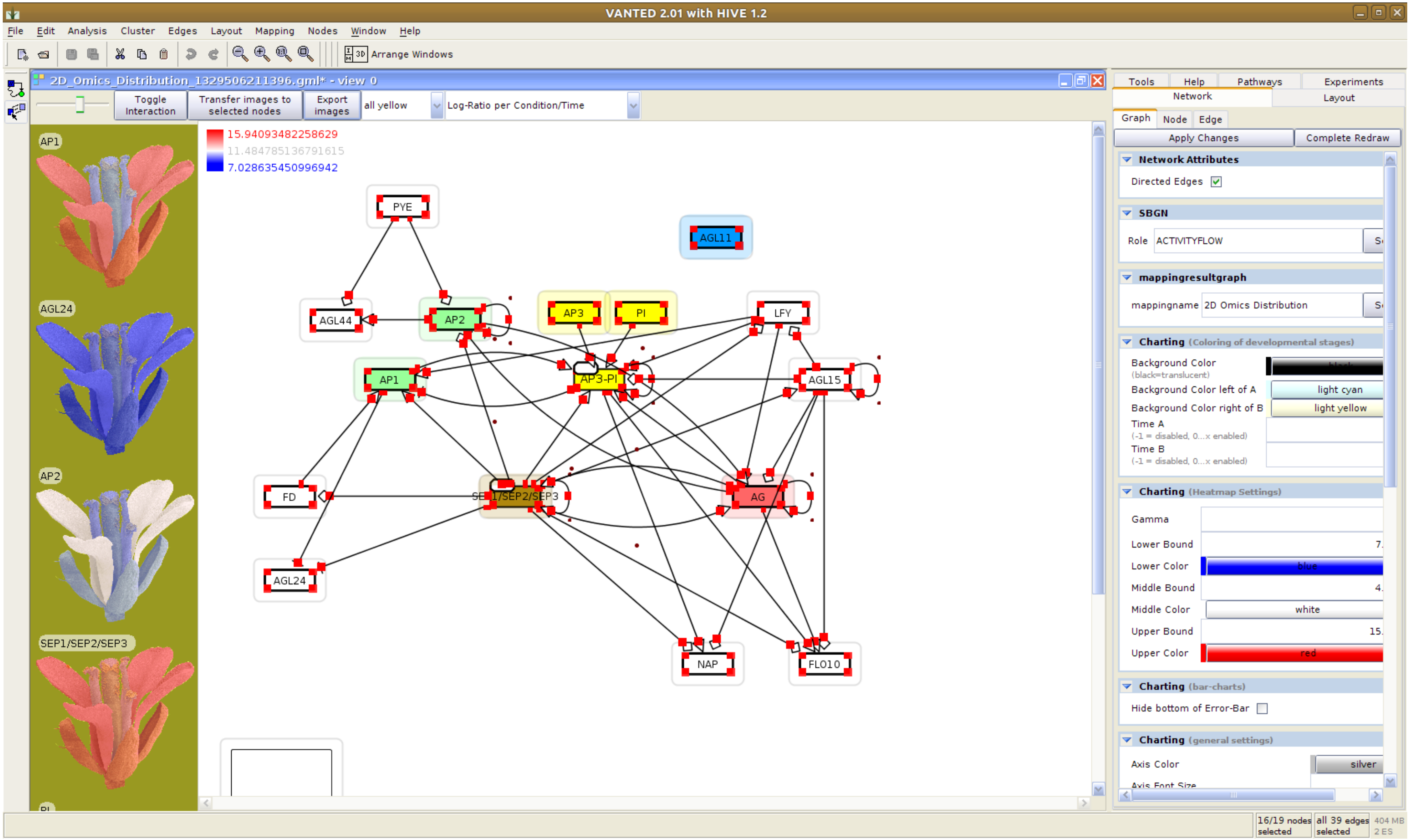

Figure 27 To integrate all images into the network nodes first select all nodes (or press <Ctrl> together with <a>) and wait until the progress bar indicates the successful generation of all images. This action may require large amounts of memory for large images or networks (400 images require about 1500MB memory).

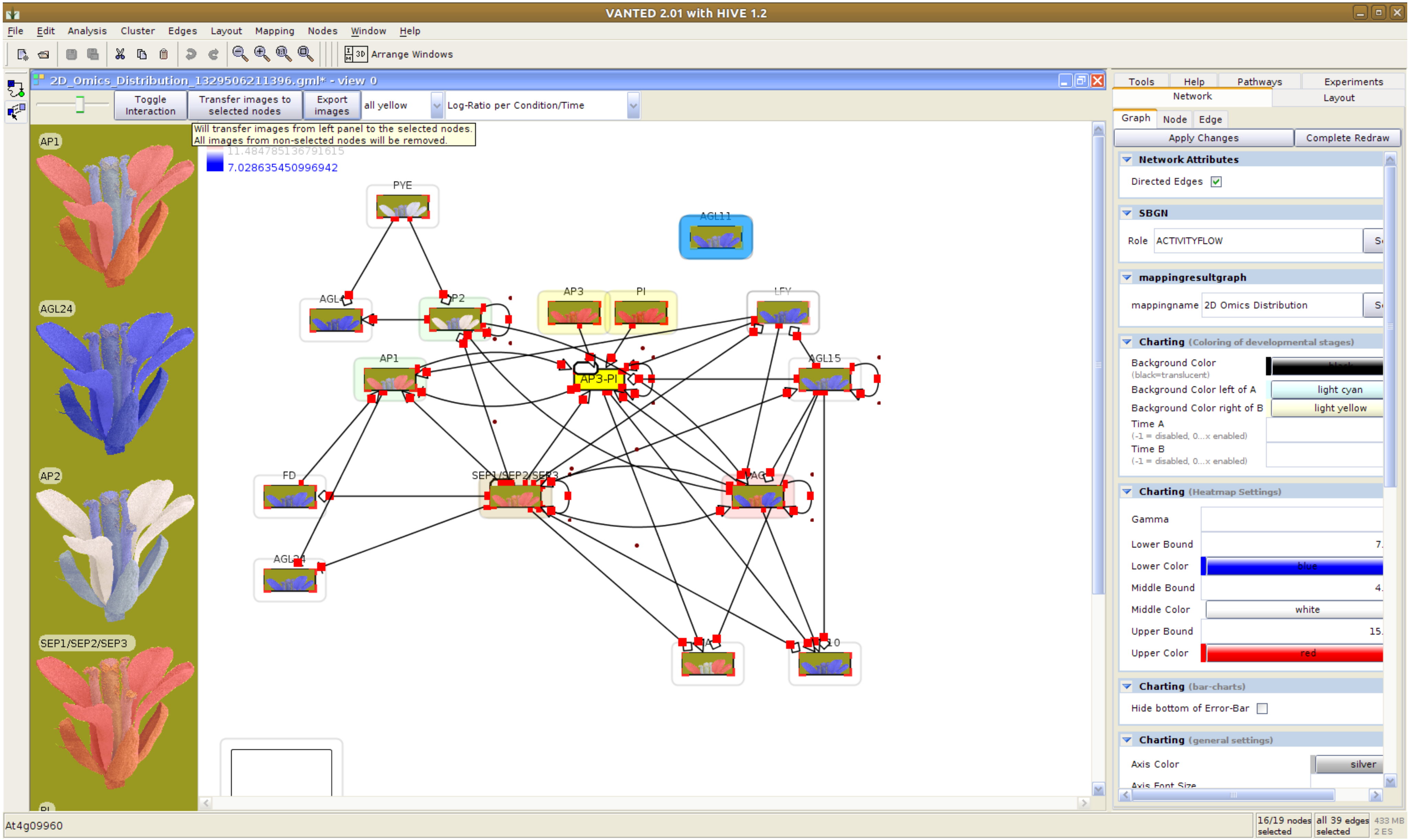

Figure 28 Integrate all generated images by clicking on button "Transfer images to selected nodes" at the top. The resolution of the integrated images depends on the size of the left imageview. If you enlarge this area using the mouse wheel (as already described in Figure 29) the resolution of the images will also be increased.

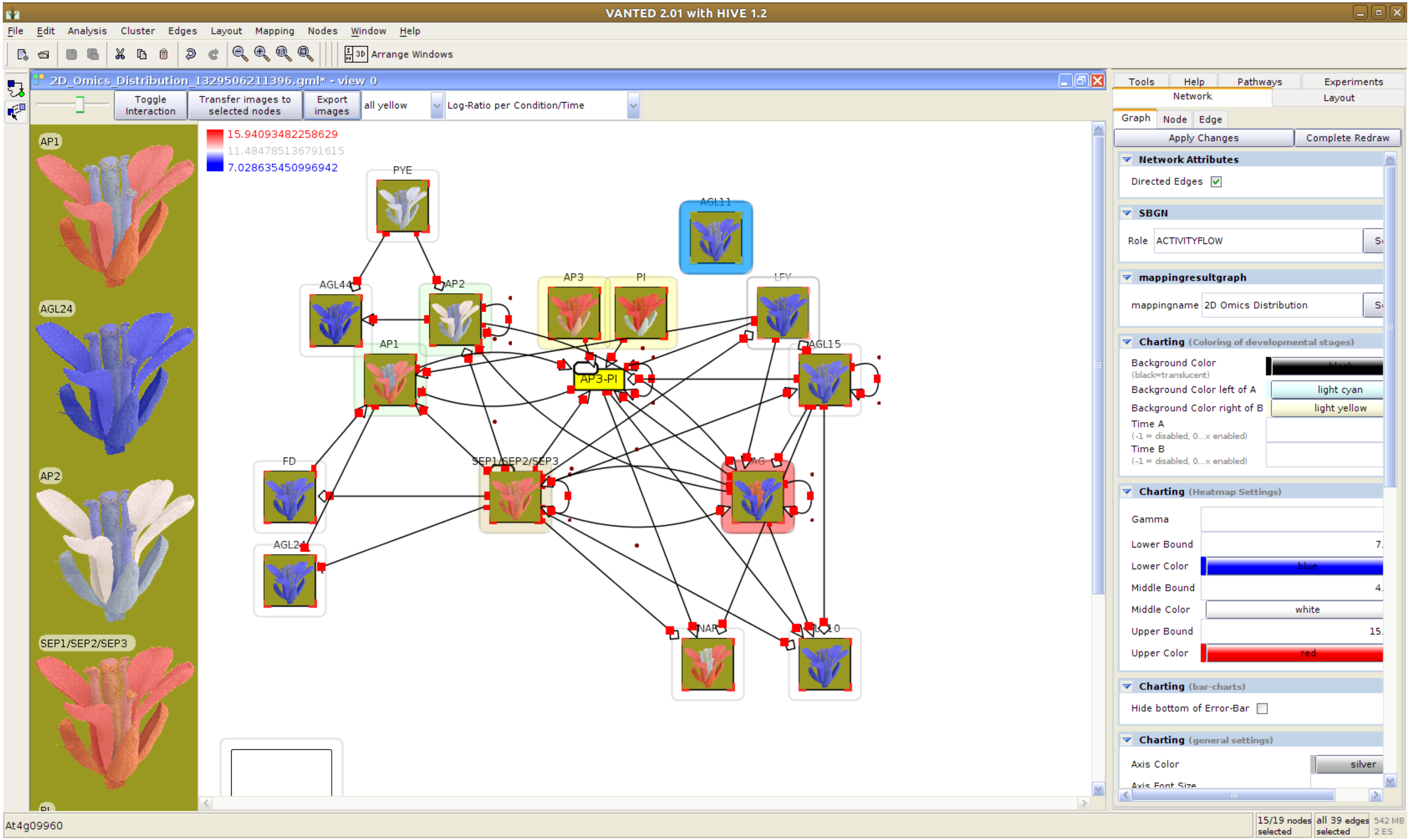

Figure 30 Adapt node sizes by dragging the corner of a node. By choosing "File" → "Create Image File" you may export the visualization as PNG, JPG, SVG, PDF or PowerPoint. Choose "File" → "Create Website" you may also create an interactive website (see the protocol of Junker et. al. 2012). If the resolution of the integrated images is too small, please enlarge the image area.

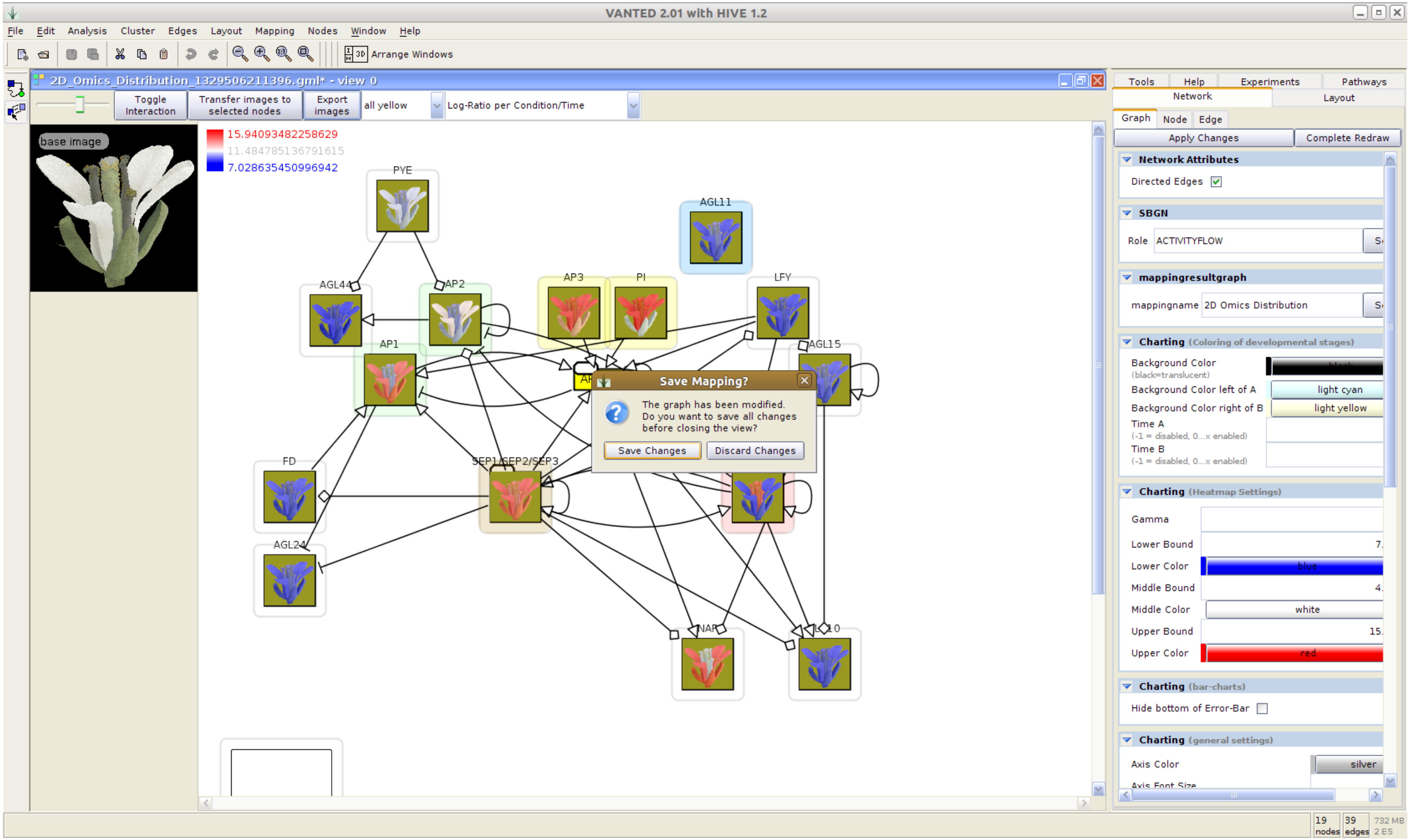

Figure 31 This represents the "Integration of colour-coded images and biological networks" step. After your exploration is finished you can close the view and select "Save changes". Please note that the images in the nodes are not stored in the network. The procedure to integrate images has to be repeated when loading the mapping again.

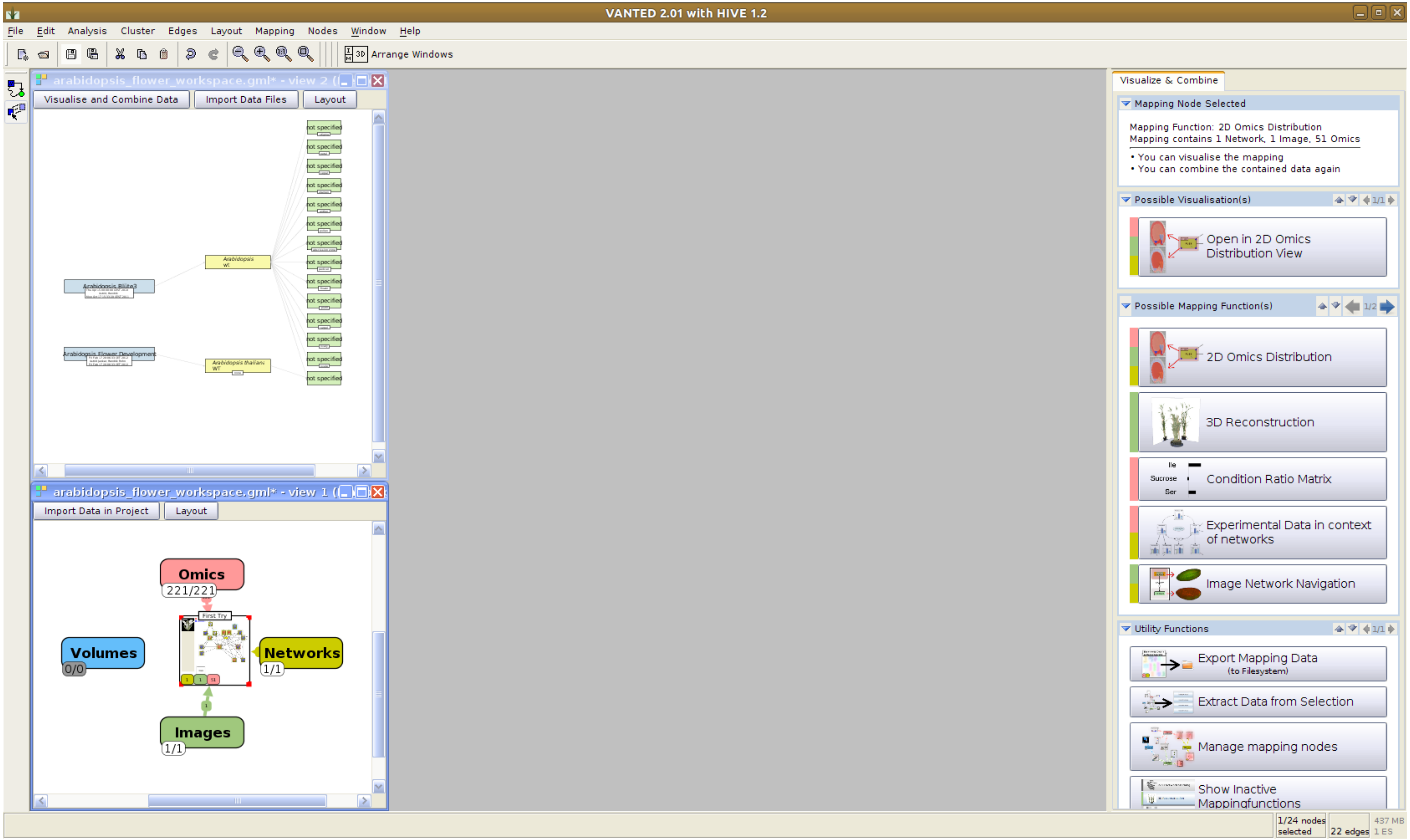

Figure 32 Don't forget to save your integrationgraph by selecting menu "File" → "Save". You may now distribute the directory you saved your integrationgraph in to any collaborators.

How to manually segment images

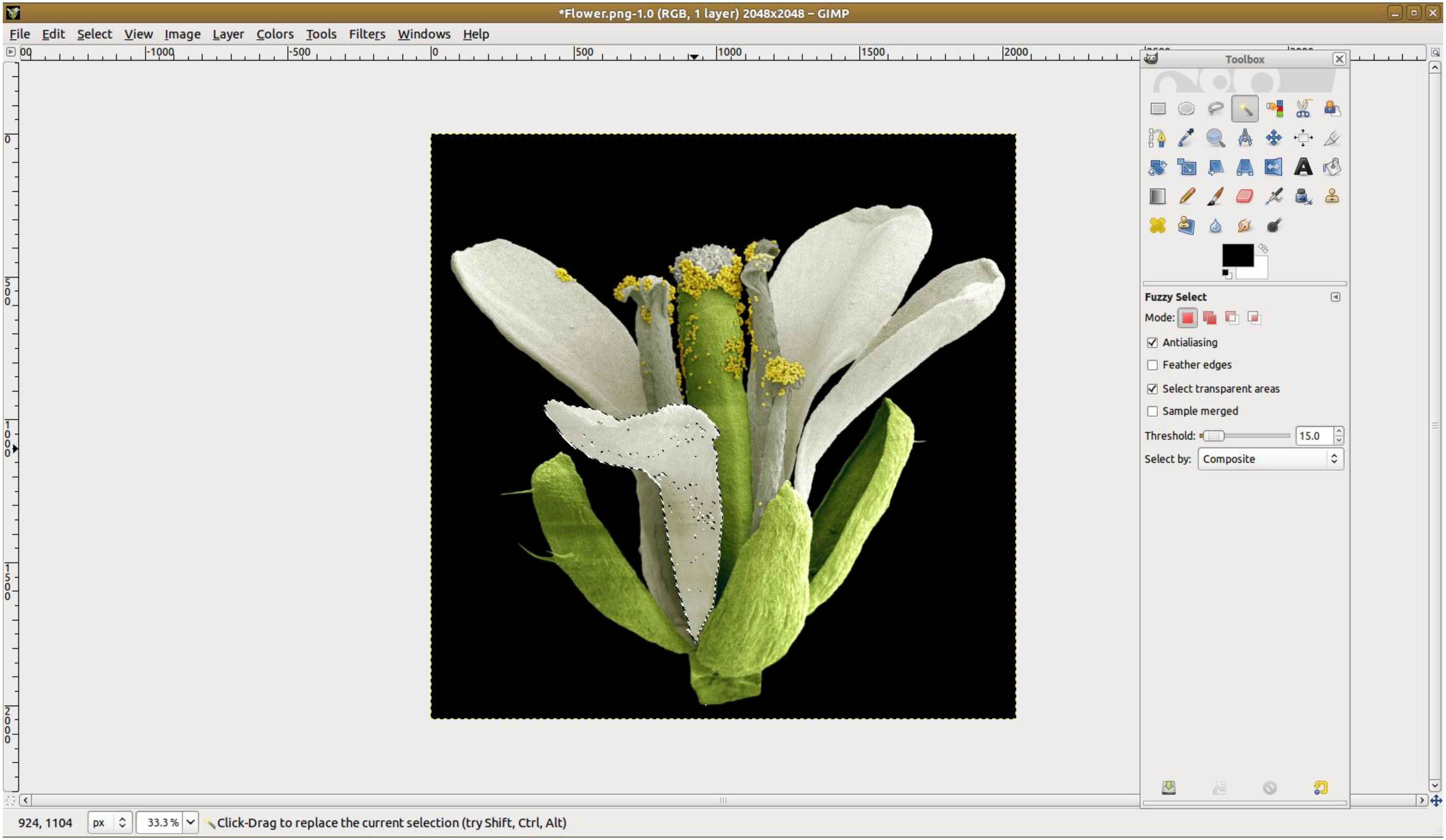

Figure 33 In order to segment an image choose your preferred image manipulation tool (here GIMP). Select the "Fuzzy Select Tool" and drag the mouse pointer in order to select a region with similar colour. Repeat this procedure while holding the <Shift> key until the selection covers your desired segment (here one petal).

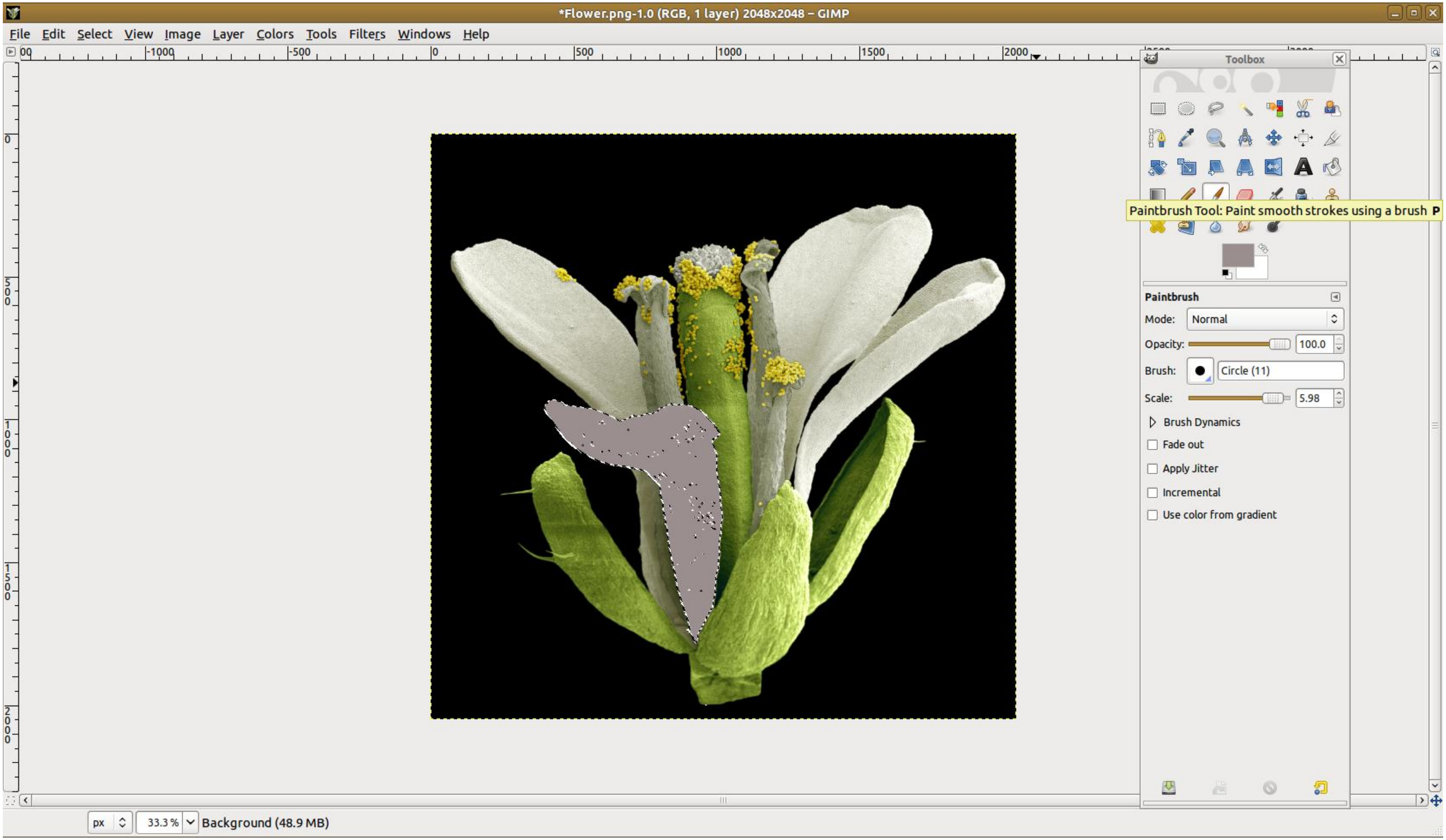

Figure 34 Choose the "Paintbrush Tool" and colourize the selected area in one colour.

Repeat the steps in Figures 32– 33 for all petals and assign the very same colour (as all belong to the organ "petal"). Repeat this procedure for all other organs and assign unique colours for each organ.

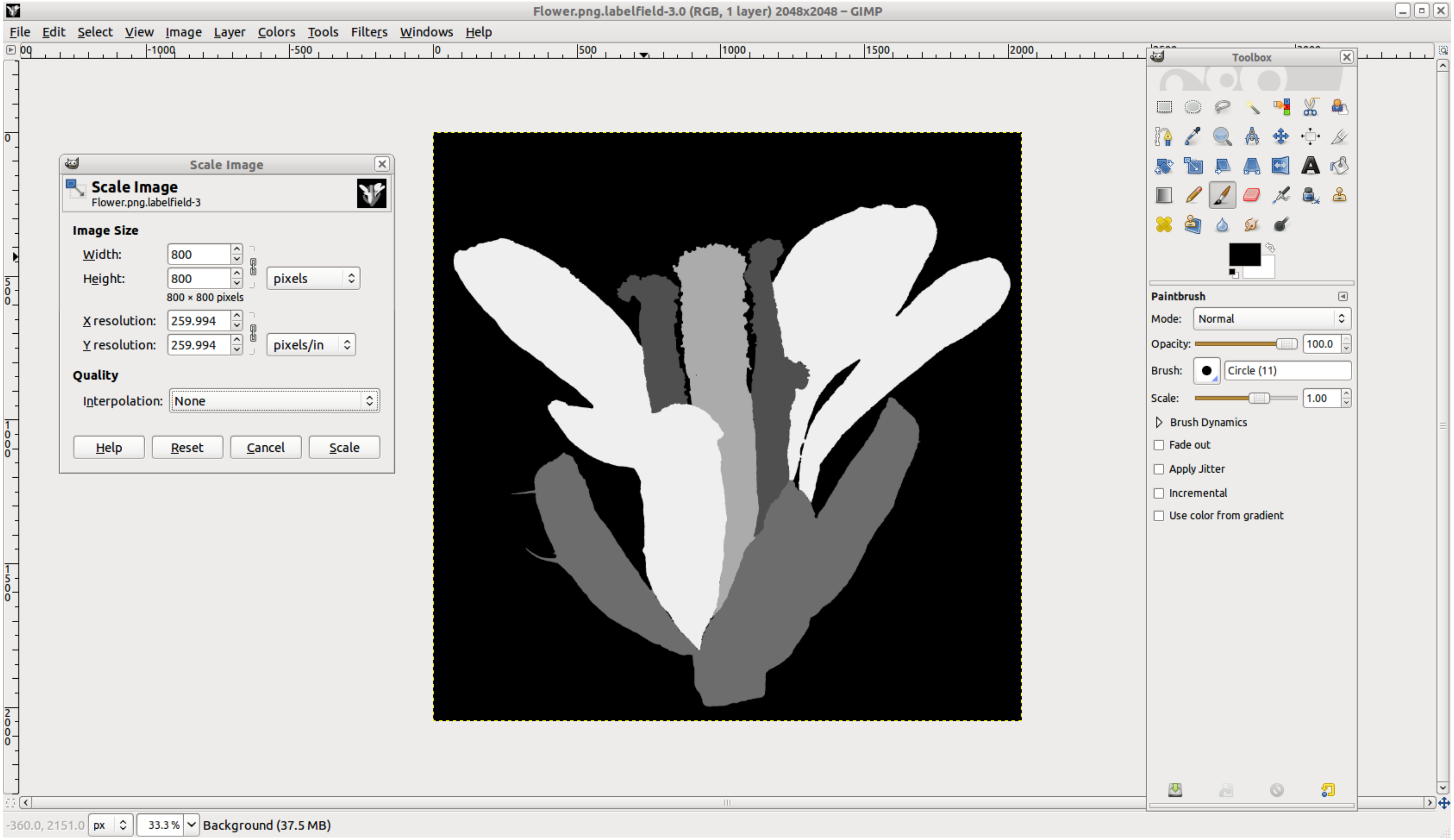

Figure 35 Set image to gray-scale ("Image" → "Mode" → "Grayscale") and save the resulting image as an image file ending with ".labelfield" in the same folder as your original image. You should consider downscaling your image, as this will reduce the processing time and memory consumption significantly (desired are <300.000 pixels). If you do so, be sure to not use any interpolation.

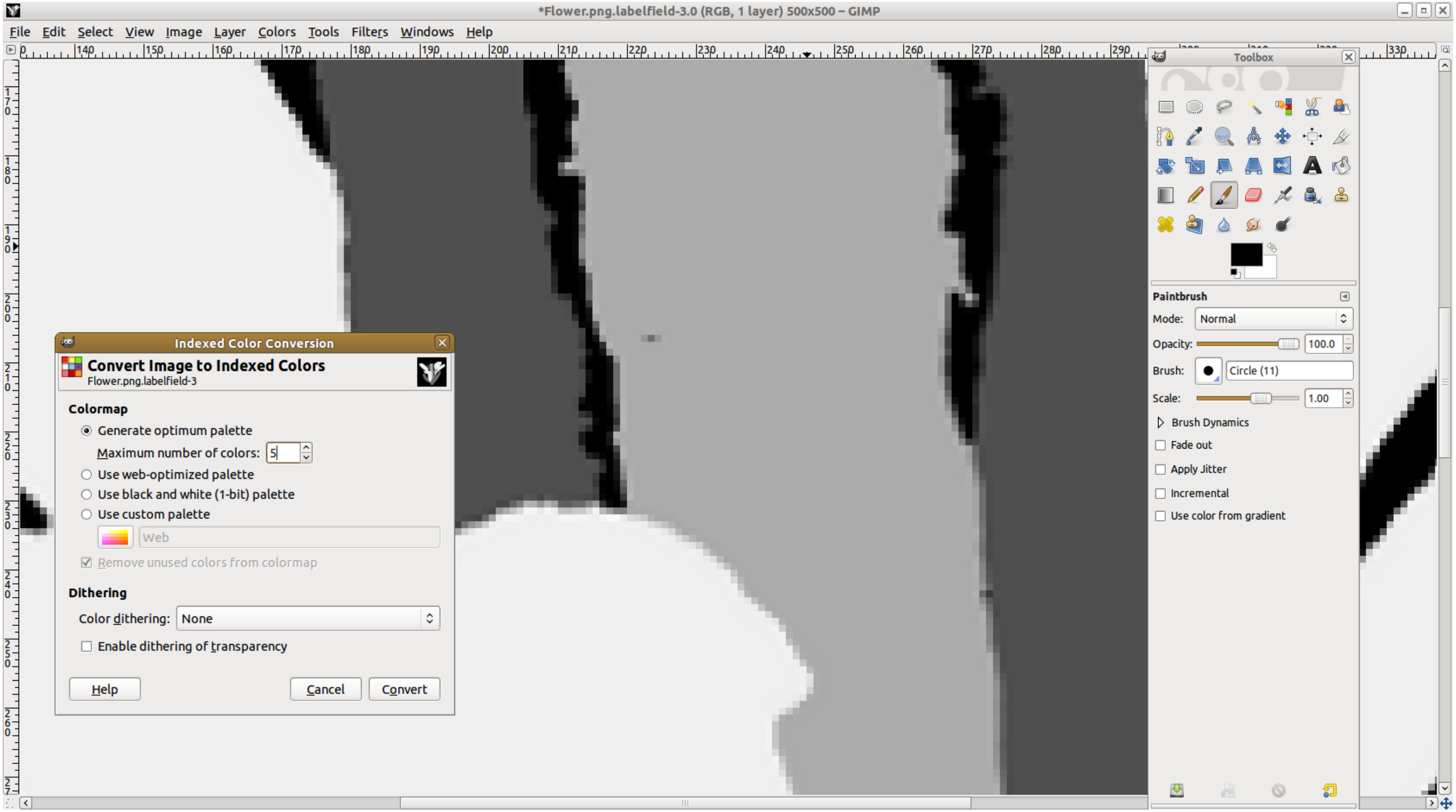

Figure 36 It is important to check the labelfield before importing it into HIVE: Be sure that the image contains only pixels with colours corresponding to a desired organ and the borders between organ are well defined. In this Figure you see the resulting image after scaling with interpolation. Please note the fuzzy organ borders. Each colour found in the image will be interpreted as a separate organ, ending up with a large list of unwanted organs. Manual correction and temporarily reducing image colours to small numbers of gray shades (see dialog) is needed to remove segmentation error.
