# Supplementary figures and images for: Visual Analysis of Transcriptome Data in the Context of Anatomical Structures and Biological Networks
Source: Front Plant Sci. 2012 Nov 15;3:252. doi: 10.3389/fpls.2012.00252 (PMC3498740; doi:10.3389/fpls.2012.00252)

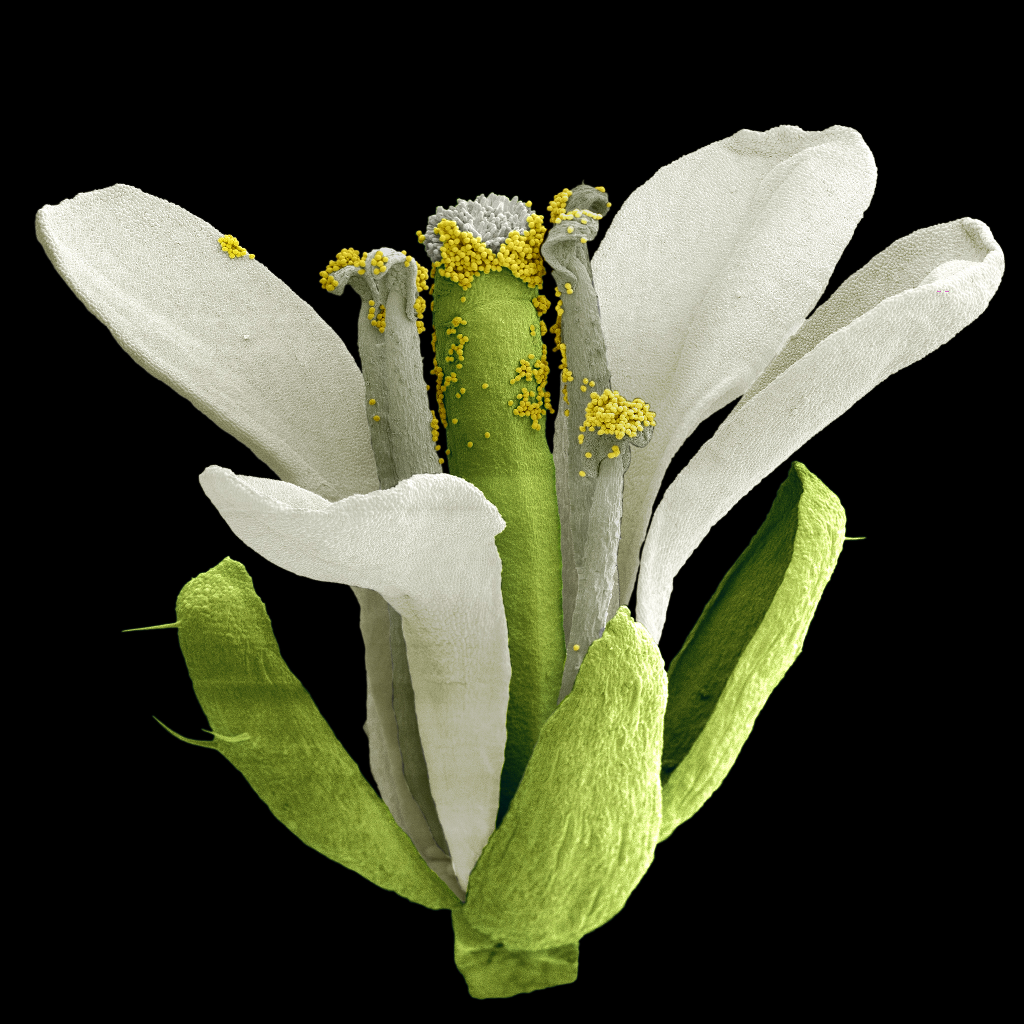

Supplement: Supplementary File S2 — Raw data for usecase 1 (ZIP; including homeotic gene-regulatory network in SBGN Activity Flows, HIVE excel template with flower expression data, Electron Microscopic image of the Arabidopsis flower and the corresponding segmented image). [file 33720_Rohn_DataSheet2.ZIP › Flower_scaled.png]

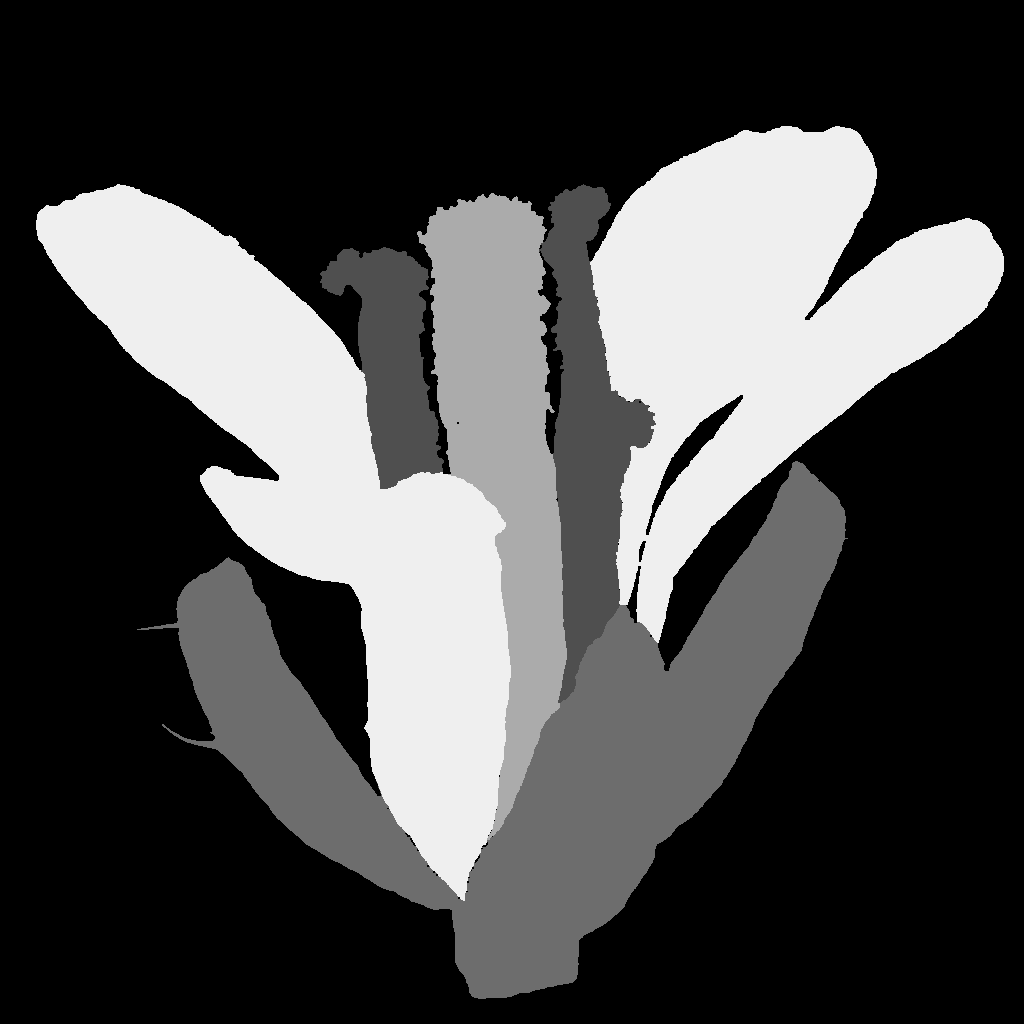

Supplement: Supplementary File S2 — Raw data for usecase 1 (ZIP; including homeotic gene-regulatory network in SBGN Activity Flows, HIVE excel template with flower expression data, Electron Microscopic image of the Arabidopsis flower and the corresponding segmented image). [file 33720_Rohn_DataSheet2.ZIP › Flower_scaled.png.labelfield]

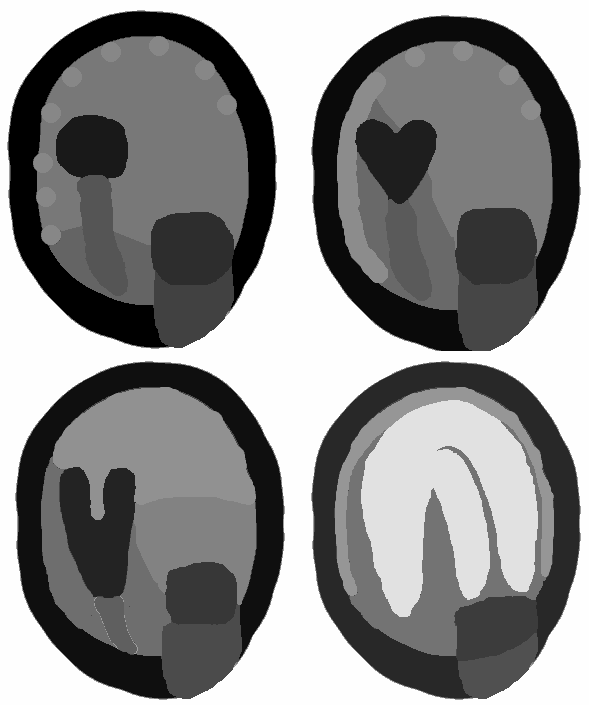

Supplement: Supplementary File S3 — Raw data for usecase 2 (ZIP; including AFLB3/LEC1 gene-regulatory network in SBGN Process Descriptions, HIVE excel template with seed expression data, schematic image of the Arabidopsis seed stages and the corresponding segmented image, high-resolution image of Figure 4A). [file 33720_Rohn_DataSheet3.ZIP › seed_dev_montage4.png]

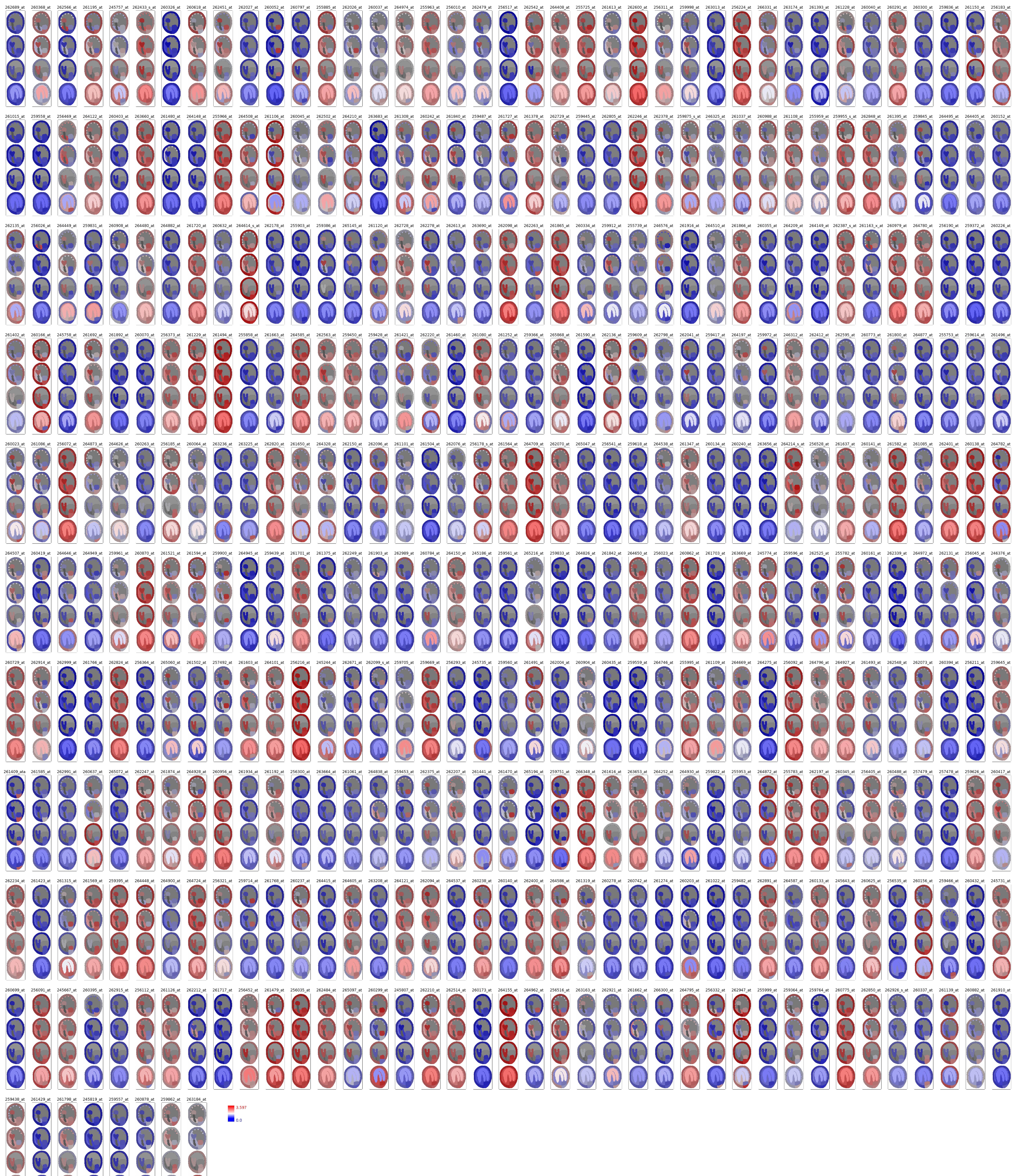

Supplement: Supplementary File S3 — Raw data for usecase 2 (ZIP; including AFLB3/LEC1 gene-regulatory network in SBGN Process Descriptions, HIVE excel template with seed expression data, schematic image of the Arabidopsis seed stages and the corresponding segmented image, high-resolution image of Figure 4A). [file 33720_Rohn_DataSheet3.ZIP › Figure 4A HR.jpg]
